# Bisphosphonates and breast cancer survival: a meta-analysis and trial sequential analysis of 81508 participants from 23 prospective epidemiological studies

**DOI:** 10.18632/aging.203395

**Published:** 2021-08-10

**Authors:** YuPeng Liu, Shu Zhao, YuXue Zhang, Justina Ucheojor Onwuka, QingYuan Zhang, XiaoDong Liu

**Affiliations:** 1 Department of Preventive Medicine, School of Public Health and Management, Wenzhou Medical University, Wenzhou, China; 2 Department of Medical Oncology, Harbin Medical University Cancer Hospital, Harbin, China; 3 Department of Microbiology, School of Public Health, Harbin Medical University Cancer Hospital, Harbin, China; 4 Department of Epidemiology, School of Public Health, Harbin Medical University, Harbin, China

**Keywords:** diphosphonates, breast neoplasms, survival, meta-analysis, trial sequential analysis

## Abstract

Background: We assessed the effect of bisphosphonates (BPs) on breast cancer (BCa) patient survival and explored how long the effect can persist after treatment.

Methods: We performed a meta-analysis and trial sequential analysis (TSA) of prospective studies including randomized controlled trials (RCTs) and cohort studies. We performed extensive sensitivity analyses to assess the robustness of the findings.

Results: Seventeen RCTs and eight cohorts with 81508 BCa patients were identified. A significant beneficial effect of BPs on BCa survival was found (RR, 0.725; 95% CI, 0.627-0.839), and the TSA results also suggested firm evidence for this beneficial effect. Both summarized results from RCTs and cohorts provided firm evidence for this effect, although the effect estimates were stronger from cohorts than RCTs (RR, 0.892; 95% CI, 0.829-0.961; 0.570; 95% CI, 0.436-0.745; respectively). This beneficial effect was confirmed for bone-metastases (RR, 0.713; 95% CI, 0.602-0.843) and postmenopausal women (RR, 0.737; 95% CI, 0.640-0.850). Importantly, our results demonstrated that this beneficial effect was retained at least 1-2 years after treatment completion (RR, 0.780; 95% CI, 0.638-0.954) and could persist for up to more than 4 years after treatment completion (RR, 0.906; 95% CI, 0.832-0.987). Extensive sensitivity analyses showed the robustness of our results. The GRADE quality of evidence was generally judged to be moderate to high.

Conclusions: The present study provides firm evidence for a significant beneficial effect of BPs on BCa survival in patients with early-stage BCa, and this effect was retained at least 1-2 years after BP treatment completion.

## INTRODUCTION

Breast cancer (BCa) is the most frequently diagnosed cancer and the leading cause of cancer deaths among females worldwide, with more than 2 million newly diagnosed cases in 2018 [1, 2]. the morbidity rates are highest in economically developed countries but are now increasing rapidly in developing countries. Although mortality from BCa has significantly declined over the past two decades, approximately half of these cases have spread as micrometastases at the time of initial diagnosis [3]. Consequently, a considerable number of patients suffer from recurrences or metastases after lesion resection, with up to approximately 70% of patients developing bone metastases [4]. Because metastatic BCa and advanced disease cannot be cured, an efficient, safe, and well-tolerated prevention strategy to improve BCa survival is therefore urgently necessary.

Bisphosphonates (BPs) are widely used for the prevention and treatment of osteoporosis and are recommended as the standard care for bone loss or skeletal-related complications caused by malignancies [5, 6]. Recently, the evidence has shown that BPs have potential anticancer properties in BCa patient survival. Nonetheless, the findings from individual studies have been inconsistent. To address this issue, several meta-analyses [7–15] have assessed the association between the risk of BCa prognosis and BP treatment; most of them have evaluated the impact of either zoledronic acid [8, 9, 12, 14] or clodronate [7, 11] treatment, and some have incorporated the results from randomised controlled trials (RCTs) with diverse control groups, including non-BP, delayed BP treatment, [9, 10, 13, 14] or denosumab treatment [8].

A recent large meta-analysis by the Early Breast Cancer Trial Collaborative Group (EBCTCG) showed a substantially significant reduction in bone metastases and BCa mortality in postmenopausal patients [16]. Then, a panel of European experts recommended that BPs should be used as part of routine clinical practice in the prevention of metastases in early BCa patients with low oestrogen (natural or induced) based on the EBCTCG meta-analysis and a consensus meeting [17]. However, BPs has not been currently approved for the adjuvant treatment of patients with early-stage BCa. In addition, there are unanswered questions such as whether the existing data are sufficient to draw firm conclusions about the effectiveness of BPs on BCa survival, as well as whether this beneficial effect persists over time.

Trial sequential analysis (TSA) is a novel method for improving the quality of information from conventional meta-analysis and providing TSA-boundaries that determine whether the evidence is reliable and conclusive. Therefore, we performed this comprehensive meta-analysis and TSA of data from prospective epidemiological studies to determine and quantify the impact of BPs on BCa survival in women with early-stage BCa. We also explored whether the effect persists, attenuates, or disappears over time. Furthermore, we evaluated the robustness of our findings with extensive sensitivity analyses.

## MATERIALS AND METHODS

### Search strategy and selection criteria

This study was registered on the PROSPERO database (CRD42014014699) and was reported according to the PRISMA guidelines. We systematically searched the electronic databases of PubMed, Embase, CENTRAL (Cochrane Central Register of Controlled Trials), and ProQuest through 25 June 2019 to identify relevant publications (detailed information on the search strategy is provided in the Supplementary Materials). We also searched ClinicalTrials.gov and Clinicaltrialsregister.eu for potentially eligible studies including completed and ongoing RCTs, which could possibly have posted their interim results online. The reference sections and citation lists of the retrieved literature, including original research articles, reviews, editorials, and letters, were reviewed for potentially relevant articles.

The inclusion criteria are as follows: (1) prospective epidemiological studies including RCTs or cohort studies that addressed the effect of BPs versus non-BP control on BCa survival among BCa patients with no evidence of any relapse or metastasis; (2) studies that reported effect estimates, such as hazard ratio (HR), relative risk ratio (RR), or odds ratio (OR), with 95% confidence intervals (CIs), or reported sufficient information to calculate these values; (3) BP users are defined as BCa patients who received BP treatment only before the occurrence of any BCa relapse or metastasis; (4) when multiple articles reporting on the same study but reporting outcomes at different time points, we used the data at the longest follow-up time in the overall meta-analysis and included all of the datasets at different follow-up time points from the same study in subgroup analyses, cumulative meta-analyses, and TSAs by follow-up duration; and (5) when no-event trials were identified, we used an empirical constant continuity correction (correction factor, 1) and we included no-event trials in the main analysis. There were no restrictions on language, sample size, or participant characteristics.

### Data extraction and quality assessment

The eligibility determination, data extraction, and quality assessment for each study were performed independently by two reviewers (YPL and YXZ). Discrepancies were resolved by discussion and consensus with another reviewer (SZ). Standard electronic forms specifically created for the present study were used to extract the data from each study. Both the maximally adjusted and unadjusted effect sizes with 95% CIs were recorded, if available. The extracted data from each study were carefully checked before performing the analyses. When necessary, we contacted the authors of studies for missing information. For RCTs, the bias risk was assessed with the domains recommended by the Cochrane collaboration tool. Trials were considered to have a high risk of bias overall (low-quality) if ≥4 of these domains were judged to have a high or unclear risk. Otherwise, they were considered to have a low risk of bias overall (high-quality). We used RevMan version 5.3 to assess the risk of bias. We assessed the quality of the cohort studies with a previously described quality-scoring system [18]. Briefly, the quality score of each cohort was presented as a percentage of the maximum score (95), and studies with a score of >60% were categorized as high-quality.

### Statistical analysis

### Meta-analysis

In this meta-analysis, the maximally adjusted effect sizes and 95% confidence intervals (CIs) were summarized using random-effects models rather than fixed-effects models to analyse the results in a conservative manner. In the main analysis, we included only the high-quality studies. Predefined subgroup meta-analyses by type of BPs, recurrence site, and menopausal status of participants in individual studies were conducted. To assess how long the beneficial effect would be maintained, we performed subgroup analyses according to the follow-up duration of RCTs, taking into account different BP treatment periods and different follow-up periods across the RCTs; we did not include cohort studies in this analysis because of the uncertain BP treatment duration. Comprehensive Meta Analysis version 2.0 (Biostat, Englewood, NJ, USA) was used for the meta-analyses and conventional cumulative meta-analyses.

We tested the between-study heterogeneity using the *Q* test and the *I*^2^ statistic. To investigate potential sources of the between-study heterogeneity, we performed subgroup and meta-regression analyses using random-effects models. To assess potential publication bias, we used funnel plots for asymmetry and formally used Begg’s rank correlation and Egger’s linear regression tests. Furthermore, we robustly adjusted for the summarized results by applying Duval and Tweedie’s trim and fill method.

### Sensitivity analysis

We conducted several prespecified sensitivity analyses. Confounding RRs were used to test whether the underlying confounders could have influenced the results. The confounding RR is defined as the ratio of the summarized results of the maximally adjusted and unadjusted data [19]. To test the potential impact of an unmeasured confounder on the results, we performed E-value analysis, which is a novel method to assess how robust the observed association is to potential unmeasured confounding [20]. By removing the most relatively weighted study, we aimed to assess its influence on the summarized estimates and to explore potential sources of heterogeneity across studies. We performed a post hoc subgroup analysis by study design. We conducted a sensitivity analysis including low-quality studies. A sensitivity analysis excluding the no-event trial [21] was conducted. We also excluded cohort studies [22, 23] in which BP use began before the diagnosis of BCa.

### Trial sequential analysis

Conventional cumulative meta-analyses are prone to producing random errors because of few data and repetitive testing of accumulated data when the required information size (RIS) has not been met [24]. We therefore performed TSA, calculated RIS and constructed trial sequential monitoring boundaries (TSMB). The RIS and TSMB were determined by event proportion in the control group, an anticipated relative risk reduction and the diversity index (*D*^2^, a heterogeneity-adjustment factor). The diversity adjusted RIS (*D*^2^-RIS) was used to reduce type II errors, and the TSMB was used to reduce type I errors. In this study, the incidence of outcome events in the control group and an anticipated relative risk reduction were estimated based on the summarized results of the included studies. TSA was conducted to maintain an overall two-sided 5% risk of type I error and a 20% risk of type II error (80% power), which is the common standard in most meta-analyses. When the *z*-curve crosses the TSMB, a firm level of evidence has been reached, and if the accumulated information size simultaneously reaches the *D*^2^-RIS, one may conclude that no further studies are needed. If the *z*-curve does not cross any of the boundaries and *D*^2^-RIS has not been reached, there is insufficient evidence to draw conclusions. When the accumulated information size reached the *D*^2^-RIS, we further conducted a post hoc sensitivity analysis with the intention to maintain a 90% power and to assess consistency and reliability of the observed effect. When several articles were published from the same study, only the data of the longest follow-up period were analysed in the TSA according to publication year, whereas in the TSA for follow-up duration, we included all of the datasets with diverse follow-up durations from the same study, from the shortest to the longest among the analysed RCTs, irrespective of the treatment period. TSA Viewer version 0.9beta (Copenhagen Trial Unit, Copenhagen, Denmark) was used for TSA.

### GRADE criteria

We used GRADE methodology to assess the quality of evidence, which was classified as high, moderate, low, or very low based on the evaluation for study design, bias risk, inconsistency, indirectness, imprecision, publication bias, and confounding bias. Summary tables were constructed with the online version of GRADEproGDT software.

## RESULTS

### Study characteristics

The flow diagram of study selection is shown in Supplementary Figure 1. Supplementary Tables 1, 2 summarize the main characteristics of the included RCTs and cohorts, respectively. A total of 27 publications [21, 25–50] from 17 RCTs were included, of which six trials [21, 39–41, 48–50] were placebo-controlled randomised trials and the other 11 trials were non-placebo designed RCTs. All of these RCTs originated from developed countries and enrolled 15586 patients with early-stage BCa, with approximately half (56.19%; 8758/15586) of the participants recorded as postmenopausal female patients at baseline. A total of 8291 BCa cases were administered intravenous zoledronic acid, [21, 25, 28, 30, 31, 33, 46, 47] oral clodronate, [34, 37, 39, 41] oral pamidronate, [42, 48] ibandronate, [43, 49] or risedronate [50]. All but three of these RCTs assessed the effect of 1 to 3 years of BP treatment on BCa survival, while Kristensen [42] evaluated the effect of 4 years of pamidronate therapy, and Coleman [31] and von Minckwitz [46] evaluated the potential effect of 5 years of zoledronic acid therapy. The sample size varied considerably with a range from 40 to 3359 participants. Across these published literatures, the mean follow-up period was 68.2 months (median: 63.3 months; range: 12 to 120 months), and 3125 outcome events were reported (BP group: 1597; control group: 1528). In one trial [21], no event occurred during the study period. The medication pattern for clodronate was more consistent than that for zoledronic acid. Among the four RCTs conducted for clodronate, patients in the intervention arms were given a dosage of 1600 mg/day of oral clodronate for two [34, 39] or three years [37, 41]. In the other eight RCTs evaluating zoledronate, patients assigned to the zoledronic acid group received 4 mg intravenously, with total doses ranging from 4 [30] to 24, [33] and the frequency of administration varied from every 3 weeks [28, 31] to every 6 months [25, 31]. Among these RCTs, 15 trials were high-quality, and two were low-quality (Supplementary Figure 2).

Among these eight cohorts included, two studies were nested case-control studies. All cohorts originated from developed countries and enrolled 66899 BCa patients, with 14731 (22.02%) of the participants recorded as BP users. The sample size varied considerably from 226 to 21190. The mean follow-up period was 78.2 months (median: 63.6 months; range: 42 to 141.6 months), and 6670 outcome events were reported (BP group: 862; control group: 5808). All cohort studies were categorized as high-quality (Supplementary Table 3).

### BPs and BCa survival

**Figure 1:**
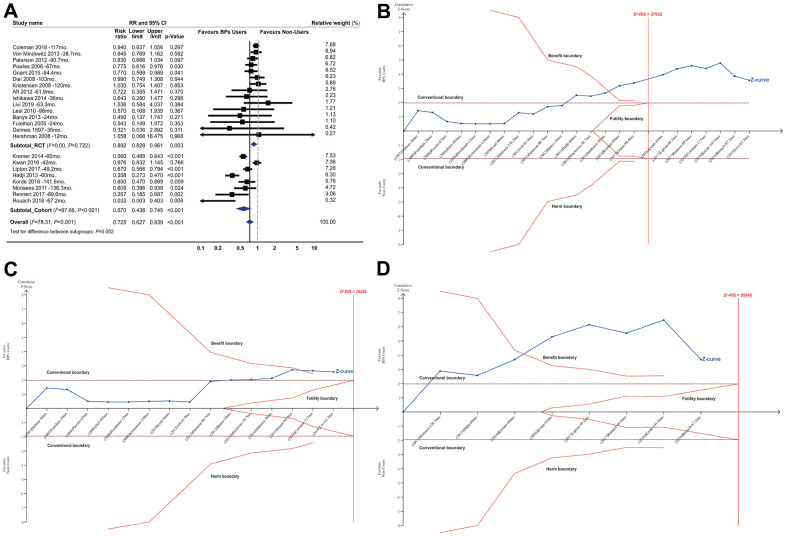
**Summarized results of bisphosphonates on breast cancer survival.** (**A**) Conventional meta-analysis by study design*; (**B**) Trial sequential analysis combining RCTs and cohorts†; (**C**) Trial sequential analysis for RCTs; (**D**) Trial sequential analysis for cohort studies. Abbreviations: BPs, bisphosphonates; CI, confidence interval; *D*^2^-RIS, the diversity adjusted required information size in trial sequential analysis; mo., months; RCTs, randomized controlled trials; RR, risk ratio; the black square represents effect size of each study; the blue diamond represents the summarized effect sizes. *Studies were ordered based on their relative weight. †The solid red vertical line represents the diversity adjusted required information size (*D*^2^-RIS). The solid red outer curves (trial sequential monitoring boundaries for benefit or harm) represent the sequential analysis thresholds for statistical significance. The solid red inner wedge curves inside the horizontal dotted brown lines represent the futility boundaries. The horizontal dotted brown lines represent the conventional thresholds for statistical significance at a constant z value of 1.96, which corresponds to a two-sided *P*-value of 0.05. The solid blue line is the cumulative z curve and represents the accumulating amount of information as studies are added, each square denoting an individual study. If the cumulative z curve crosses the *D*^2^-RIS line, it represents that the *D*^2^-RIS has been currently accrued. If the cumulative z curve crosses the benefit or harm boundary, it represents conclusive evidence in favour of bisphosphonate users or non-users respectively. If the cumulative z curve crosses the futility boundaries, then it would be extremely unlikely that the addition of future studies would demonstrate any significant effect. In the panel B (combining RCTs and cohort studies), the actually cumulative sample size in this analysis has currently excessed the *D*^2^-RIS and the cumulative z curve crossed the benefit boundary. In the panels of C and D (for RCTs and cohort studies respectively), the *D*^2^-RIS has not been reached but the cumulative z curve crossed the benefit boundary for both RCTs and cohort studies.

BP treatment significantly improved the survival of the patients with early-stage BCa, with a summarized RR of 0.725 (95% CI, 0.627 to 0.839; *P*<0.001; 81508 participants; Table 1 and Figure 1A). In the TSA, the *D*^2^-RIS was 37932 participants. The TSA showed that the cumulative *z* curve had crossed the trial sequential monitoring boundary (TSMB) for the beneficial effects of BPs, and the actually cumulative sample size (ACSS) currently exceeded the *D*^2^-RIS with a summarized RR of 0.700 (TSA corrected 95% CI, 0.589 to 0.832; *P*<0.001), implying that there is firm evidence for an overall protective effect of BPs over controls in women with primary BCa (Figure 1B). We then conducted an exploratory TSA to maintain 90% power and found that the cumulative *z* curve crossed the TSMB for beneficial effects and that the ACSS also exceeded the *D*^2^-RIS, also providing firm evidence for the beneficial effects of BPs (Supplementary Figure 3). The GRADE quality of evidence was judged to be moderate (Supplementary Table 4). Subgroup analyses by study design showed that beneficial effects were stronger in cohorts than RCTs, with summarized RRs of 0.570 (95% CI, 0.436 to 0.745; *P*<0.001; 66899 participants) and 0.892 (95% CI, 0.829 to 0.961; *P*=0.003; 14609 participants), respectively. TSA showed that the cumulative *z* curve crossed the TSMB for beneficial effects in both RCTs (Figure 1C) and cohorts (Figure 1D), although approximately 70% of the *D*^2^-RIS was accrued, also implying substantial evidence for the beneficial effect. The GRADE quality of evidence was judged to be high for RCTs and moderate for cohorts.

**Table 1:** Summarized results of effects of bisphosphonate treatment versus no bisphosphonates (control) on survival of breast cancer patients.

**Subgroups**	**No. of datasets**	**ACSS**	**Incidence in control group (%)**	**Pooled effect estimates**		***I*^2^ (*P*-value)**	*D*^2^ (%)*	***D*^2^-RIS**	**Crosses TSMB / FB**
				**RR (95% CIs), *P*-value**	**TSA adjusted 95% CIs, *P*-value**				
**Overall**	23	81850	7190/58989 (12.19)	**0.725 (0.627, 0.839), <0.001**	**0.700, (0.589, 0.832), <0.001**	78.31 (<0.001)	94.26	37932	**TSMB**
**Study Design**									
RCTs	15	14609	1382/6821 (20.25)	**0.892 (0.829, 0.961), 0.003**	**0.923, (0.856, 0.995), 0.015**	0.00 (0.722)	0.00	20228	**TSMB**
Cohorts	8	66899	5808/52168 (11.13)	**0.570 (0.436, 0.745), <0.001**	**0.510, (0.318, 0.817), 0.0002**	87.66(<0.001)	96.28	95945	**TSMB**
**Follow-up Duration (year) (Only RCTs)**									
Treatment duration	5	4738	472/2376 (19.84)	0.930 (0.817, 1.058), 0.270	0.905, (0.418, 1.960), 0.300	0.00 (0.437)	62.79	36198	NO
Post-treatment 1-2	8	8820	882/3891 (22.67)	**0.780 (0.638, 0.954), 0.016**	**0.774, (0.604, 0.992), 0.012**	48.91 (0.057)	83.94	11983	**TSMB**
Post-treatment 3-4	4	3345	288/1669 (17.26)	**0.718 (0.585, 0.881), 0.001**	0.798, (0.386, 1.653), 0.160	27.98 (0.244)	76.43	14424	NO
Post-treatment >4	7	9913	1014/4965 (20.42)	**0.906 (0.832, 0.987), 0.024**	0.905, (0.798, 1.027), 0.029	0.00 (0.597)	28.55	18292	NO
**Recurrence Sites**									
Bone metastases	12	42970	1900/27002 (7.04)	**0.713 (0.602, 0.843), <0.001**	**0.705, (0.527, 0.943), 0.019**	61.47 (0.003)	91.50	48175	**TSMB**
Nonskeletal metastases	9	20634	897/10895 (8.23)	0.883 (0.768, 1.014), 0.078	0.903, (0.684, 1.192), 0.155	35.88 (0.131)	61.10	91020	NO
Loco-regional recurrence	10	38074	2692/25357 (10.62)	0.887 (0.771, 1.020), 0.093	**0.639, (0.380, 1.075), 0.013**	11.56 (0.336)	91.94	21094	**FB**
Contralateral recurrence	11	39009	972/26171 (3.72)	**0.775 (0.651, 0.922), 0.004**	**0.722, (0.606, 0.861), <0.001**	0.00 (0.500)	0.00	9181	**FB**
**Menopausal Status**									
Post	12	30764	1891/23462 (8.06)	**0.737 (0.640, 0.850),< 0.001**	**0.737, (0.607, 0.895), 0.002**	38.27 (0.086)	78.41	28195	**TSMB**
Non-post	6	4178	530/1949 (27.17)	0.992 (0.864, 1.139), 0.913	NA†	5.46 (0.382)	NA	NA†	NA
**Type of BPs (Only RCTs)**									
Zoledronic acid	7	5716	666/2863 (23.24)	**0.892 (0.805, 0.988), 0.029**	0.920, (0.790, 1.072), 0.079	0.00 (0.620)	0.00	15730	NO
Clodronate	3	4682	387/2340 (16.54)	**0.846 (0.736, 0.971), 0.017**	0.891, (0.555, 1.431), 0.215	0.00 (0.415)	50.74	25661	NO

Abbreviations: ACSS, the actually cumulative sample size; BPs, bisphosphonates; CI, confidence interval; *D*^2^, diversity; FB, futility boundary; HR, hazard ratio; *D*^2^-RIS, the diversity adjusted required information size; NA, not applicable; TSA, trial sequential analysis; TSMB, trial sequential monitoring boundary.

*D^2^, diversity, defined as the relative variance reduction when the meta-analysis model is changed from random effects models into fixed effect models.

^†^The information size for the subgroup trial sequential analyses was too low to require futility boundaries.

### The longevity of the beneficial effect

**Figure 2:**
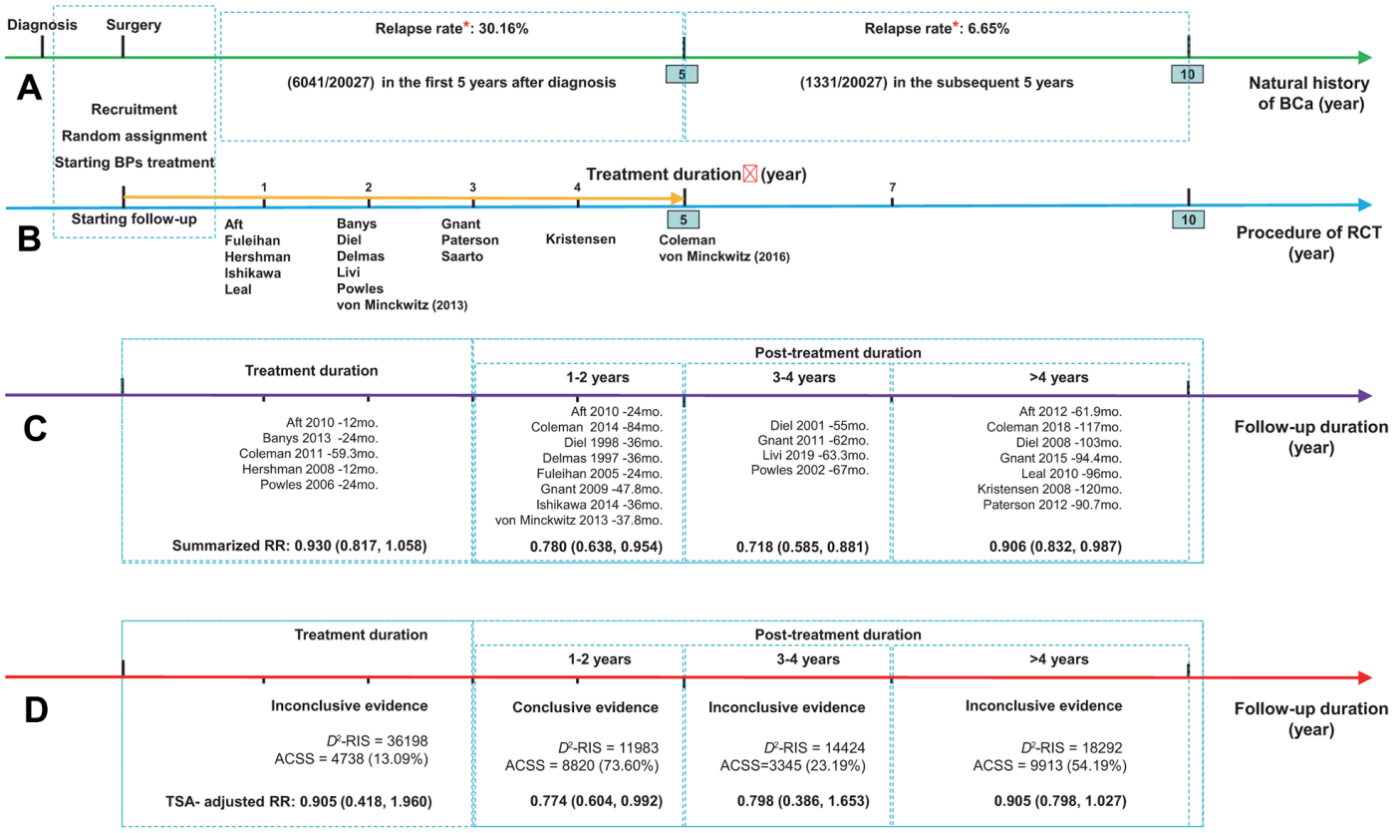
**Summarized results for the longevity of the effect of bisphosphonates on breast cancer survival.** (**A**) The natural history of breast cancer after diagnosis, presented as green arrow; (**B**) The procedure of RCT presented as blue arrow, and golden arrow represents the duration of adjuvant treatment with bisphosphonates; (**C**) The results from conventional subgroup analyses by follow-up duration; (**D**) The results from trial sequential analyses of each subgroup by follow-up duration. *Data regarding the relapse rate of breast cancer were obtained from the Surveillance, Epidemiology, and End Results (SEER) program of the National Cancer Institute (Cheng et al. 2012). Abbreviations: ACSS, the actually cumulative sample size; BCa, breast cancer; BPs, bisphosphonates; *D*^2^-RIS, the diversity adjusted required information size; RCT, randomized controlled trial; RR, hazard ratio; TSA, trial sequential analysis.

**Figure 3:**
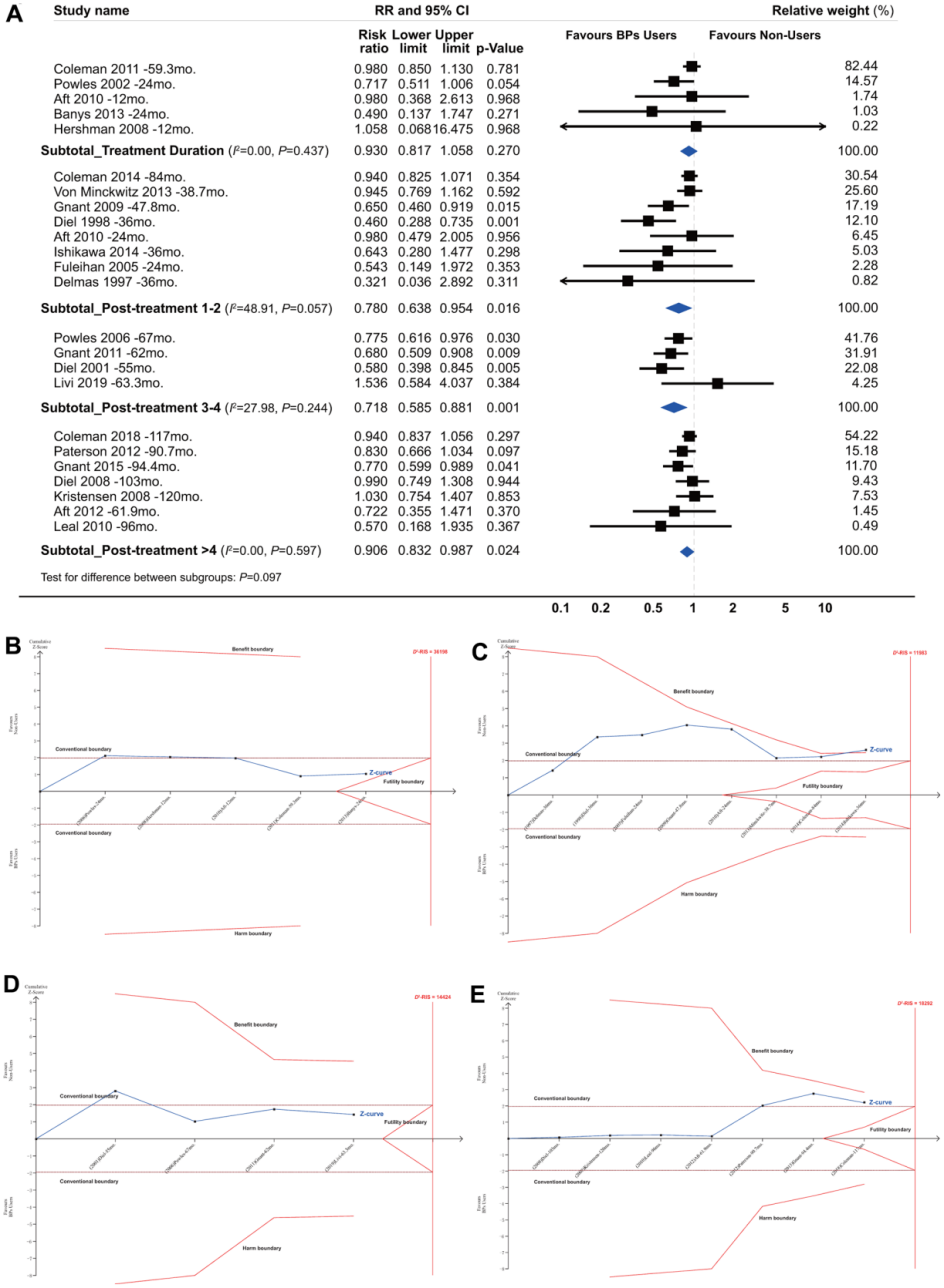
**Summarized results for the longevity of the effect from conventional subgroup analysis and trial sequential analysis by follow-up duration.** *(**A**) Conventional subgroup meta-analysis; (**B**) Trial sequential analysis for subgroups of bisphosphonate treatment duration; (**C**) Post-treatment 1-2 years; (**D**) Post-treatment 3-4 years and (**E**) Post-treatment >4 years. *In these analyses, we included only RCTs and excluded all the cohort studies, because that the duration of bisphosphonate treatment was not identical for bisphosphonate users in each individual cohort study and none of them reported a clear and explicit treatment duration.

Figures 2, 3 summarized the results for the longevity of the beneficial effect. Conventional subgroup analyses showed that BP treatment improved BCa patient survival during the follow-up periods after the end of BP treatment, and the beneficial effect was maintained until more than 4 years after BP treatment (RR, 0.780; 95% CI, 0.638 to 0.954; *P*=0.016; 8820 participants; RR, 0.718; 95% CI, 0.585 to 0.881; *P*=0.001; 3345 participants; RR, 0.906; 95% CI, 0.832 to 0.987; *P*=0.024; 9913 participants; for post-treatment 1-2 years, 3-4 years, and >4 years, respectively; Figure 3A), although this was not observed for the treatment duration (HR, 0.930; 95% CI, 0.817 to 1.058; *P*=0.270; 4738 participants). In the TSA, however, only the finding for post-treatment 1-2 years was confirmed (Figure 3B–3E). The cumulative *z* curve had just crossed the benefit TSMB, although 73% of the *D*^2^-RIS (11983) was accrued, suggesting firm evidence for a carryover beneficial effect for up to 1-2 years after treatment completion. For subgroups of post-treatment 3-4 or >4 years, TSA-corrected effects did not reach statistical significance, although the cumulative z curve for the later subgroup crossed the conventional boundary.

The TSA according to follow-up duration showed that the cumulative *z* curve crossed the benefit TSMB when the follow-up duration reached 42 months with a final summarized HR of 0.716 (TSA corrected 95% CI, 0.618 to 0.830; *P*<0.001), supporting the evidence for this carryover beneficial effect (Supplementary Figure 4A). Notably, this beneficial effect appeared to be much more obvious during the follow-up period from 42 to 67 months. Meanwhile, the *z* curve was far from the futility boundary, suggesting that further trials are unlikely to overturn this result. A sensitivity analysis, which included only the dataset with the longest follow-up period when multiple datasets were published from the same study, showed identical results (Supplementary Figure 4B).

### Subgroup analyses by recurrence site

**Figure 4:**
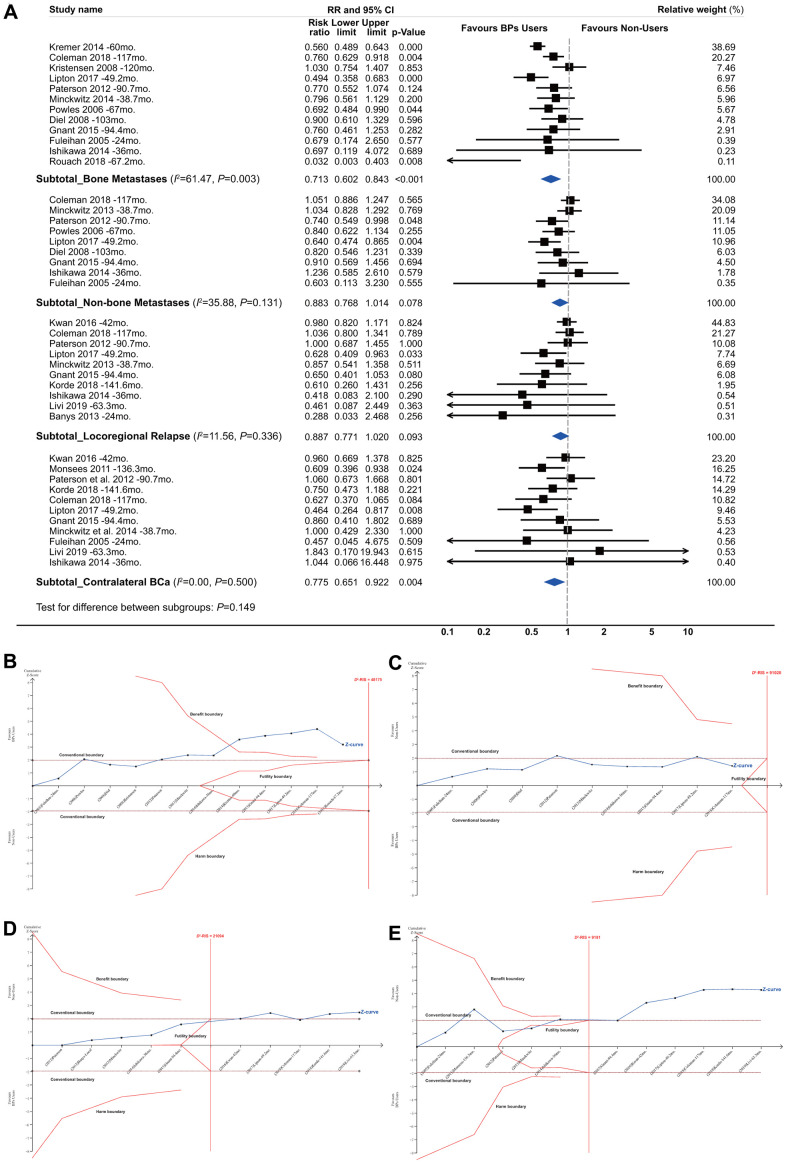
**Summarized results of bisphosphonates and breast cancer survival by recurrence site.** (**A**) Conventional subgroup meta-analysis by recurrence site; (**B**) Trial sequential analysis for subgroups of bone metastases; (**C**) Non-skeletal distant metastases; (**D**) Local-regional relapses and (**E**) contralateral breast cancer.

When analyses were restricted to bone metastases, the pooled RR was 0.713 (95% CI, 0.602 to 0.843; *P*<0.001; 42970 participants; Figure 4A). The TSA showed that 89.20% of the *D*^2^-RIS of 48175 patients was accrued, but the cumulative *z* curve crossed the beneficial TSMB with a summarized effect estimate of 0.705 (TSA corrected 95% CI, 0.527 to 0.943; *P*=0.019), suggesting firm evidence for a protective effect of BPs against bone metastases (Figure 4B). There was no significant effect for the incidence of non-skeletal distant metastases and local-regional relapses according to either conventional meta-analysis or TSA (Figure 4C, 4D). TSA showed that the cumulative *z* curve crossed the futility boundary (FB), suggesting firm evidence for no effect of BPs on local-regional relapses. For contralateral BCa, the conventional meta-analysis showed a summarized RR of 0.775 (95% CI, 0.651 to 0.922; *P*=0.004; 39009 participants), but the TSA showed that the cumulative *z* curve crossed the FB (Figure 4E), suggesting that estimates from conventional meta-analysis may represent false-positive results (Type I error).

### Subgroup analyses by menopause status

**Figure 5:**
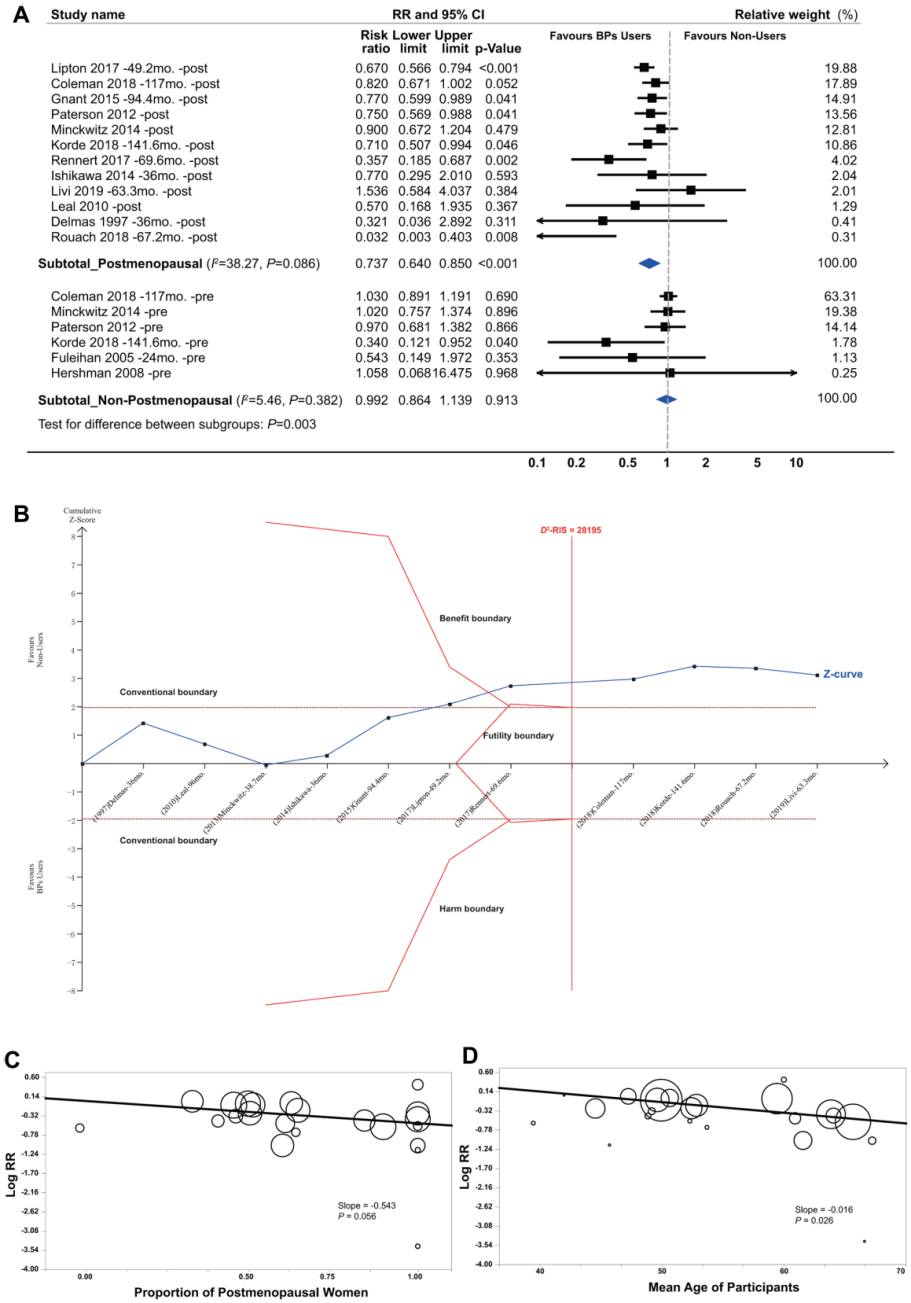
**Summarized results of bisphosphonates and breast cancer survival by menopause status.** (**A**) Conventional subgroup meta-analysis by menopause status; (**B**) Trial sequential analysis of the postmenopausal subgroup; (**C**) Meta-regression analysis based on the proportion of postmenopausal women and (**D**) the mean age of participants in individual studies. In the panels of (**C**, **D**) circles indicate individual studies, and the size of the circle is proportional to the relative weight that the study has in calculating the summary effect estimate.

Subgroup analyses showed a significant improvement in survival for postmenopausal BCa patients (RR, 0.737; 95% CI, 0.640 to 0.850; *P*<0.001; 30764 participants) but not for non-postmenopausal women (Figure 5A). For postmenopausal women, TSA provided firm evidence for beneficial effects, given that the ACSS exceeded the *D*^2^-RIS and that the cumulative *z* curve crossed the beneficial TSMB with a summarized RR of 0.737 (TSA-corrected 95% CI, 0.607 to 0.895; *P*=0.002; Figure 5B). For non-postmenopausal women, TSA was not conducted because of insufficient information size. Interestingly, meta-regression analyses revealed a borderline significant correlation between the reported effect sizes and the proportion of postmenopausal women in individual studies (slope=-0.543, *P*=0.057; Figure 5C), suggesting a trend between a stronger beneficial effect with an increasing number of postmenopausal women enrolled in the original trials. This was also supported by the findings of meta-regression analyses by the mean age of participants in the individual studies (Figure 5D).

### Sensitivity analyses

In general, our results were largely robust to sensitivity analyses. Confounding RRs demonstrated that the summarized results of the unadjusted datasets did not significantly differ from the results of the maximally adjusted data (Supplementary Table 5). Moreover, confounding RRs indicated that the summarized results were more conservative for the maximally adjusted data than the unadjusted data. E-values demonstrated that the beneficial effect of BPs on BCa survival, especially bone metastases and postmenopausal patients, appears to be very robust (Supplementary Table 6). Thus, a hypothetical unmeasured confounder needs to be very strongly associated with both BCa survival and BP treatment, and at the same time, this potential confounder must be frequent to be able to fully explain the observed beneficial effect. Examining the RCTs and cohorts separately, the beneficial effect remained significant for BCa survival, bone metastases and post-menopausal women, although the magnitude was attenuated for RCTs relative to cohorts (Supplementary Table 7). Another sensitivity analysis excluding the study with the most relative weight did not substantially change the summarized results (Supplementary Table 8). Sensitivity analyses including trials [38, 46] with an overall high risk of bias did not substantially change the results (Supplementary Table 9). Sensitivity analyses excluding the no-event trial [21] or those cohorts [22, 23] without a clear definition of post-diagnosis BP use did not substantially change the summarized results (Supplementary Tables 10, 11).

### Between-study heterogeneity and publication bias

In general, there was obvious between-study heterogeneity, which was mainly from the cohort studies. The between-study heterogeneity among RCTs was very small or nil, whereas it was very obvious for cohort studies. Findings from the meta-regression analyses (Supplementary Figure 5) revealed that the heterogeneity was possibly attributed to differences in the proportion of mean age of patients (*P*=0.026) and postmenopausal women (*P*=0.056) but not to sample size (*P*=0.486), number of BP users (*P*=0.902), outcome events (*P*=0.439) or follow-up duration (*P*=0.454). We found no evidence for publication bias (Supplementary Figure 6 and Supplementary Table 12).

## DISCUSSION

For the first time, we combined the data from prospective studies including RCTs and cohorts and confirmed the positive beneficial effect of BPs on BCa survival using the TSA method. The present study provides firm evidence for the beneficial effect of BPs on BCa survival, especially for bone metastases and postmenopausal patients. Importantly, our results demonstrated that this observed effect was retained for at least 1-2 years after treatment completion. Subgroup analyses revealed a statistically non-significant 9.5% improvement in BCa survival in the treatment duration, a significant 22.6% improvement for 1 to 2 years after treatment completion, a non-significant 20.2% improvement for 3 to 4 years after treatment completion, and a borderline significant 9.5% improvement for more than 4 years after treatment completion, which suggested that the beneficial effects may persist for up to 4 years after the treatment completion. Interestingly, this carryover effect is similar to the effect of tamoxifen on BCa survival [51, 52]. Given that only a proportion of the *D*^2^-RIS were accrued, further trials are needed. The results from eight ongoing RCTs with a total of 14286 participants that focus on the association between BPs and BCa survival are expected, and our systematic review has to be updated when these additional results are available.

In addition, the findings indicated that the benefit of BP treatment seemed to diminish during the follow-up period longer than 4 years after completion of the treatment. The diminution of the beneficial effect may be explained by a significant decrease in BP concentrations in the body. BP concentrations in the urinary excretion sharply decreased beyond 4 years after completion of the BP treatment, although it was still detectable up to 8 years after the end of treatment [53]. It is therefore plausible that BPs cannot reach the concentration required to exert a significant beneficial effect more than 4 years after treatment completion. On the other hand, the overall relapse rate of BCa is markedly higher in the first 5 years after diagnosis than in the subsequent 5 years (30.16% vs. 6.65%), according to the Surveillance Epidemiology and End Results (SEER) data from the National Cancer Institute of the United States [54]. Thus, BPs could exert a meaningfully beneficial effect during this period with a high relapse rate, and logically, the effect would tend to diminish along with a decreasing relapse rate because it may be more difficult to obtain a similarly high reduction when the risk of outcome events is relatively low. Of further interest is whether re-treatment with BPs 3 to 4 years after completion of the first round of medication would maintain a long-term equally beneficial effect thereafter. If confirmed in future studies, this could be critical for current clinical practice and adjuvant BP treatment to improve survival in patients with early stage BCa.

Our present study confirmed the results of the EBCTCG study and provided firm evidence for the beneficial effect of BPs on BCa survival, although the overall beneficial effect might be primarily from firm evidence about bone metastases. Bone is the most common metastasis site in patients with BCa [55]. The bone microenvironment plays an important role in metastasis from BCa [32]. Circulating tumour cells attracted to bone can remain dormant state for years and can evade the effects of adjuvant chemotherapies [56]. The re-activated tumour cells can subsequently either activate osteoclasts to resorb bone and cause bone metastases or potentially initiate nonskeletal relapses [57]. Therefore, for patients with early-stage BCa, it is of great importance to efficiently decrease the risk of bone metastases. We noted a distinct difference in the effects of BPs between postmenopausal and non-postmenopausal patients. We demonstrated that postmenopausal BCa patients had a much stronger beneficial effect from BPs than non-postmenopausal patients. Although the underlying biological mechanisms remain unclear, an antagonistic effect of BPs and oestrogen on altering the bone marrow microenvironment has been proposed as a potential mechanism [58]. Our findings combined with the results of the EBCTCG study suggest that the observed beneficial effect of BPs is more robust in a depleted-oestrogen environment.

Recently, an increasing body of biological mechanisms has supported the notion that BPs may modify the tumour cell microenvironment and directly affect tumour cells through a variety of pathways, including inhibition of osteolysis [59] and the release of bone-derived growth factors, [60] inhibition of protein prenylation, [61] and angiogenesis inhibition, [62] enhanced immune surveillance by activation of gamma delta T cells, [63] direct inhibition of tumour cell growth, proliferation, adhesion and invasion, and induction of apoptosis [64]. Another potential antitumor effect is that BPs may modify the bone microenvironment to inhibit the viability of residual tumour cells, including disseminated and circulating tumour cells, which has been shown to be strongly associated with the risk of BCa relapse or metastasis [65].

Given the global use of BPs among millions of women over the past two decades, it can be concluded that adverse effects related to BPs are relatively low [66]. Commonly reported side-effects are mild, whereas serious side-effects including osteonecrosis of the jaw, renal toxicity, and oesophageal cancer are rare. However, whether these serious side-effects are attributable to BPs alone remains inconclusive. Additionally, regarding osteonecrosis of the jaw, increasing awareness, regular dental screening, and maintaining good oral health during treatment are expected to reduce the risk [67]. Another concern is the potential side effects of long-term (>5 years) BP treatment; however, few studies have addressed the long-term (>5 years) side-effects of BP use [66]. Based on the current evidence, the benefits of BP treatment for 1 to 5 years significantly outweigh the potential harm from these drugs. If these results are confirmed in future confirmatory RCTs, new concerns arise: what is the optimal procedure of BP treatment, including dosing, frequency, and duration, for patients with early-stage BCa, whether the beneficial effects of intermittent or less frequent dosing BP treatment is not inferior to the standard procedure used currently, and whether a more frequent dosing BP treatment (at the cancer dose not the osteoporosis dose) provides additional oncological benefits. It could be speculated that the lack of a significant beneficial effect for the treatment duration may be due to a suboptimal dose of BPs; in other words, increasing the BP dosage or frequency may result in an earlier beneficial effect [68]. To our knowledge, there is only one ongoing RCT [69] addressing this issue that aimed to compare the effects of 2 years versus 5 years of zoledronate treatment in patients with early primary BCa. Additional well-designed trials are still necessary.

BPs should have a substantial and extensive public health benefit in the improvement of BCa survival, since BPs are already widely prescribed for the prevention and treatment of osteoporosis or cancer-induced bone loss, particularly in developed countries [17, 55], and an increasing number of BP users worldwide are expectable.

The major strengths of this study were the application of TSA and the inclusion of all of the currently available prospective epidemiological studies, including RCTs and cohort studies, in our study. To our knowledge, this is the first meta-analysis using TSA to assess the effect of BPs on BCa survival. TSA, taking into consideration the required information size, the accumulated information size, and the effect size, is more conservative and reliable and probably more accurate because it allows for repetitive testing of accumulating data. We included a total of 81508 early-stage BCa patients enrolled in the analysed studies, including 22521 BP users and 9491 events, which added reliability to our findings. The results of the RCTs and cohort studies are consistent, even though there is obvious heterogeneity between study designs. RCTs tend to evaluate the effect of BPs under ideal conditions among highly selected BCa patients, whereas cohort studies evaluate the effect in real-world settings. Therefore, it is important that new therapies be evaluated using RCTs and assessed using prospective cohort studies before interpreting the real-world effects. Additionally, a high consistency of the results across diverse statistical models and extensive sensitivity analyses and the moderate to high overall GRADE quality of evidence added further reliability and robustness to our findings.

However, several limitations of this study should be considered. First, various dosages and durations of BP treatment used in the individual studies could have contributed to the obvious heterogeneity. Second, multiple testing could have affected our results since we conducted post hoc subgroup analyses by follow-up duration and menopausal status and our findings from these subgroup analyses should be interpreted with caution. Despite being post hoc analyses, the findings of subgroup analyses by follow-up duration raised interesting hypotheses: whether the benefits of BPs can exert a carryover beneficial effect for up to 4 years after the treatment completion and whether re-treatment with BPs 3 to 4 years after completion of the first round of medication would maintain a long-term, equally beneficial effect thereafter. Even though we performed the TSA to correct for these random errors, sparse data, and repetitive testing, the results suggest there is insufficient information to confirm this hypothesis. Thus, a confirmatory large sample size RCT is required to explore and confirm this hypothesis, especially if the findings are from subgroup analyses. Finally, we could not explore whether the beneficial effect of bisphosphonates differs across different molecular subtypes of breast cancer due to the lack of available data.

In summary, this study provides firm evidence for a significant beneficial effect of BPs on BCa survival in patients with early-stage BCa, and this effect could persist at least 1-2 years after the completion of BP treatment. Several unanswered questions remain, including what is the optimal procedure of BP treatment for women with primary early-stage BCa, which type of BPs would be better, whether the beneficial effects persist or attenuate with time, or become more pronounced if a second round of treatment with BPs is given, and which group of women may derive the greatest benefit from BP treatment.

## Supplementary Material

Supplementary Materials

Supplementary Figures

Supplementary Table 1

Supplementary Table 2

Supplementary Table 3

Supplementary Table 4

Supplementary Tables 5 and 6

Supplementary Table 7

Supplementary Table 8-12
