# Supplementary material for: Bisphosphonates and breast cancer survival: a meta-analysis and trial sequential analysis of 81508 participants from 23 prospective epidemiological studies
Source: Aging (Albany NY). 2021 Aug 10;13(15):19835–66. doi: 10.18632/aging.203395 (PMC8386537; doi:10.18632/aging.203395)
Supplement: Supplementary Materials [file aging-13-203395-s001.pdf]

SUPPLEMENTARY MATERIALS

Search strategy

PubMed

#1. (((breast[MeSH Terms]) OR breast[Title/Abstract]) OR mammary glands[Title/Abstract])

#2. ((((((((((((neoplasms[MeSH Terms]) OR neoplasms[Title/Abstract]) OR neoplasm[Title/Abstract]) OR cancers[Title/Abstract]) OR cancer[Title/Abstract]) OR tumours[Title/Abstract]) OR tumour[Title/Abstract]) OR malignancies[Title/Abstract]) OR malignancy[Title/Abstract]) OR neoplasias[Title/Abstract]) OR neoplasia[Title/Abstract]) OR carcinomas[Title/Abstract]) OR carcinoma[Title/Abstract])

#3. #1 AND #2

#4. ((((((((((((breast neoplasms[MeSH Terms]) OR breast neoplasms[Title/Abstract]) OR breast neoplasm[Title/Abstract]) OR breast tumours[Title/Abstract]) OR breast tumour[Title/Abstract]) OR breast cancers[Title/Abstract]) OR breast cancer[Title/Abstract]) OR breast carcinomas[Title/Abstract]) OR breast carcinoma[Title/Abstract]) OR human mammary neoplasms[Title/Abstract]) OR human mammary neoplasm[Title/Abstract]) OR human mammary carcinomas[Title/Abstract]) OR human mammary carcinoma[Title/Abstract])

#5. #3 OR #4

#6. ((((((((((((((((((diphosphonates[MeSH Terms]) OR diphosphonates[Title/Abstract]) OR diphosphonate[Title/Abstract]) OR bisphosphonates[Title/Abstract]) OR bisphosphonate[Title/Abstract]) OR alendronate[Title/Abstract]) OR clodronic acid[Title/Abstract]) OR etidronic acid[Title/Abstract]) OR ibandronic acid[Title/Abstract]) OR pamidronate[Title/Abstract]) OR risedronic acid[Title/Abstract]) OR zoledronic acid[Title/Abstract]) OR alendron*[Title/Abstract]) OR clodron*[Title/Abstract]) OR etidron*[Title/Abstract]) OR pamidron*[Title/Abstract]) OR ibandron*[Title/Abstract]) OR risedron*[Title/Abstract]) OR zoledron*[Title/Abstract])

#7. #5 AND #6

#8. ((((((((((((((Randomized Controlled Trial[Publication Type]) OR Controlled Clinical Trial[Publication Type]) OR Randomized Controlled Trials as Topic[MeSH Terms]) OR Controlled Clinical Trials as Topic[MeSH Terms]) OR controlled trial[Title/Abstract]) OR clinical trial[Title/Abstract]) OR randomized controlled trial[Title/Abstract]) OR Controlled Clinical Trial[Title/Abstract]) OR randomized[Title/Abstract]) OR randomised[Title/Abstract]) OR plsacebo[Title/Abstract]) OR randomly[Title/Abstract])
#9. (((((Cohort Studies[MeSH Terms]) OR Cohort Studies[Title/Abstract]) OR Cohort Study[Title/Abstract]) OR cohorts[Title/Abstract]) OR cohort[Title/Abstract])
#10. #8 OR #9
#11. #7 AND #10

EMBASE

#1. 'breast cancer':ti,ab,kw OR 'breast tumour':ti,ab,kw OR 'breast caicinoma':ti,ab,kw OR 'breast neoplasm':ti,ab,kw

#2. diphosphonates:ti,ab,kw OR diphosphonate:ti,ab,kw OR bisphosphonates:ti,ab,kw OR bisphosphonate:ti,ab,kw OR alendron*:ti,ab,kw OR clodron*:ti,ab,kw OR etidron*:ti,ab,kw OR pamidron*:ti,ab,kw OR ibandron*:ti,ab,kw OR risedron*:ti,ab,kw OR zoledron*:ti,ab,kw OR 'zoledronic acid':ti,ab,kw

#3. #1 AND #2

#4. 'randomized controlled trial':ti,ab,kw OR 'controlled clinical trial':ti,ab,kw OR 'controlled trial':ti,ab,kw OR 'controlled study':ti,ab,kw OR 'clinical trial':ti,ab,kw OR placebo:ti,ab,kw OR randomized:ti,ab,kw OR randomly:ti,ab,kw

#5. 'cohort studies':ti,ab,kw OR 'cohort study':ti,ab,kw OR cohorts:ti,ab,kw OR cohort:ti,ab,kw
#6. #4 OR #5

#7. #3 AND #6

CENTRAL (Cochrane Central Register of Controlled Trials)

#1. MeSH descriptor: [Breast Neoplasms] explode all trees

#2. (breast cancer):ti, ab, kw OR (breast tumour):ti, ab, kw OR (breast carcinoma):ti, ab, kw OR (breast neoplasm):ti, ab, kw

#3. #1 OR #2

#4. MeSH descriptor: [Diphosphonates] explode all trees

#5. (diphosphonates): ti, ab, kw OR (diphosphonate): ti, ab, kw OR (bisphosphonates): ti, ab, kw OR (bisphosphonate): ti, ab, kw

#6. (alendron*): ti, ab, kw OR (clodron*): ti, ab, kw OR (etidron*): ti, ab, kw OR (pamidron*): ti, ab, kw OR (ibandron*): ti, ab, kw OR (risedron*): ti, ab, kw OR (zoledron*): ti, ab, kw

#7. #4 OR #5 OR #6

#8. #3 AND #7

ProQuest
#1. mesh(breast neoplasms) OR mainsubject(breast neoplasms) OR ab(breast cancer) OR ab(breast tumour) OR ab(breast carcinoma) OR ab(breast neoplasm)

#2. mesh(diphosphonates) OR mainsubject(biphosphonates) OR ab(diphosphonates) OR ab(diphosphonate) OR ab(biphosphonates) OR ab(biphosphonate)

#3. #1 AND #2

#4. mesh(Randomized Controlled Trials as Topic) OR mesh(controlled clinical trials as topic) OR ab(Randomized Controlled Trial) OR ab(controlled clinical trial) OR ab(controlled trial) OR ab(clinical trial) OR ab(placebo) OR ab(randomized) OR ab(randomly)

#5. mesh(cohort studies) OR mainsubject(cohort study) OR ab(cohort study) OR ab(cohort)

#6. #4 OR #5

#7. #3 AND #6
