# Supplementary material for: Bisphosphonates and breast cancer survival: a meta-analysis and trial sequential analysis of 81508 participants from 23 prospective epidemiological studies
Source: Aging (Albany NY). 2021 Aug 10;13(15):19835–66. doi: 10.18632/aging.203395 (PMC8386537; doi:10.18632/aging.203395)
Supplement: Supplementary Figures [file aging-13-203395-s002.pdf]

SUPPLEMENTARY FIGURES

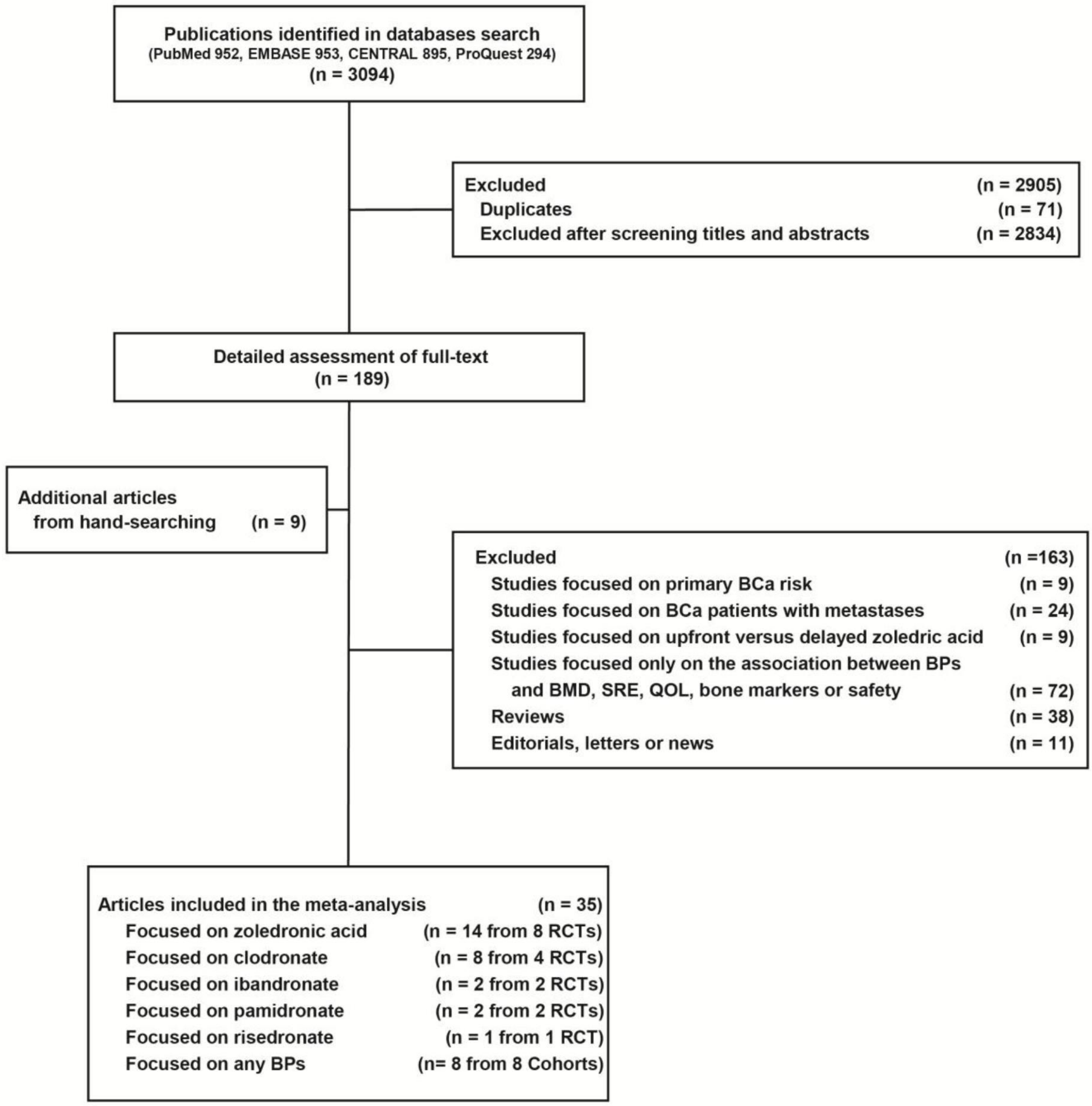

Supplementary Figure 1. Flow diagram of study selection. BCa, breast cancer; BMD, bone mineral density; BPs, bisphosphonates; QOL, quality of life; RCTs, randomized controlled trials; SRE, skeletal related event.

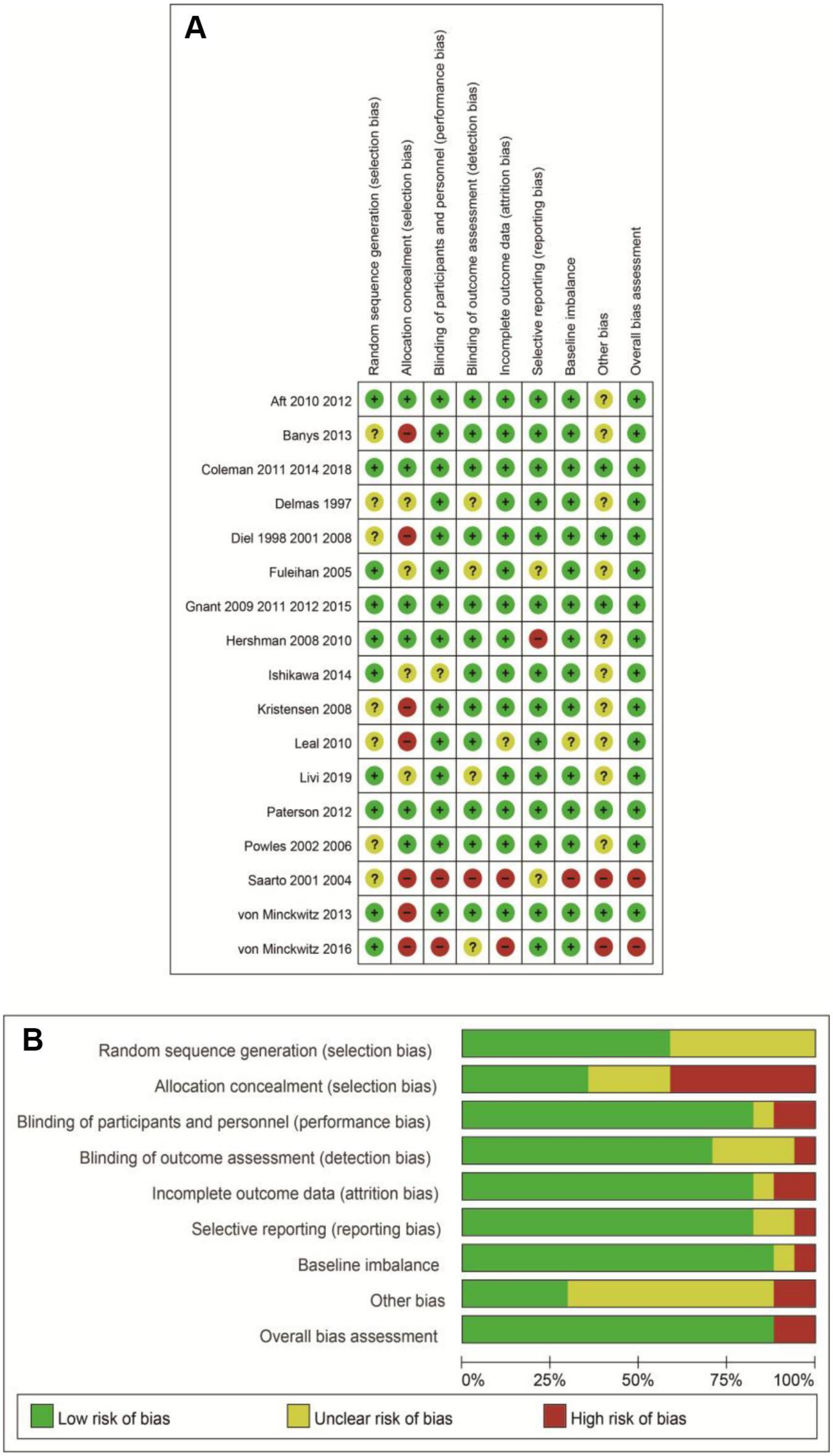

Supplementary Figure 2. Risk of bias summary displaying review authors' judgments about each risk of bias domain for each included study. (**A**) Judgements about each risk of bias item for each included study; (**B**) Judgements about each risk of bias item presented as percentages across all included studies. Studies were listed alphabetically by author name.

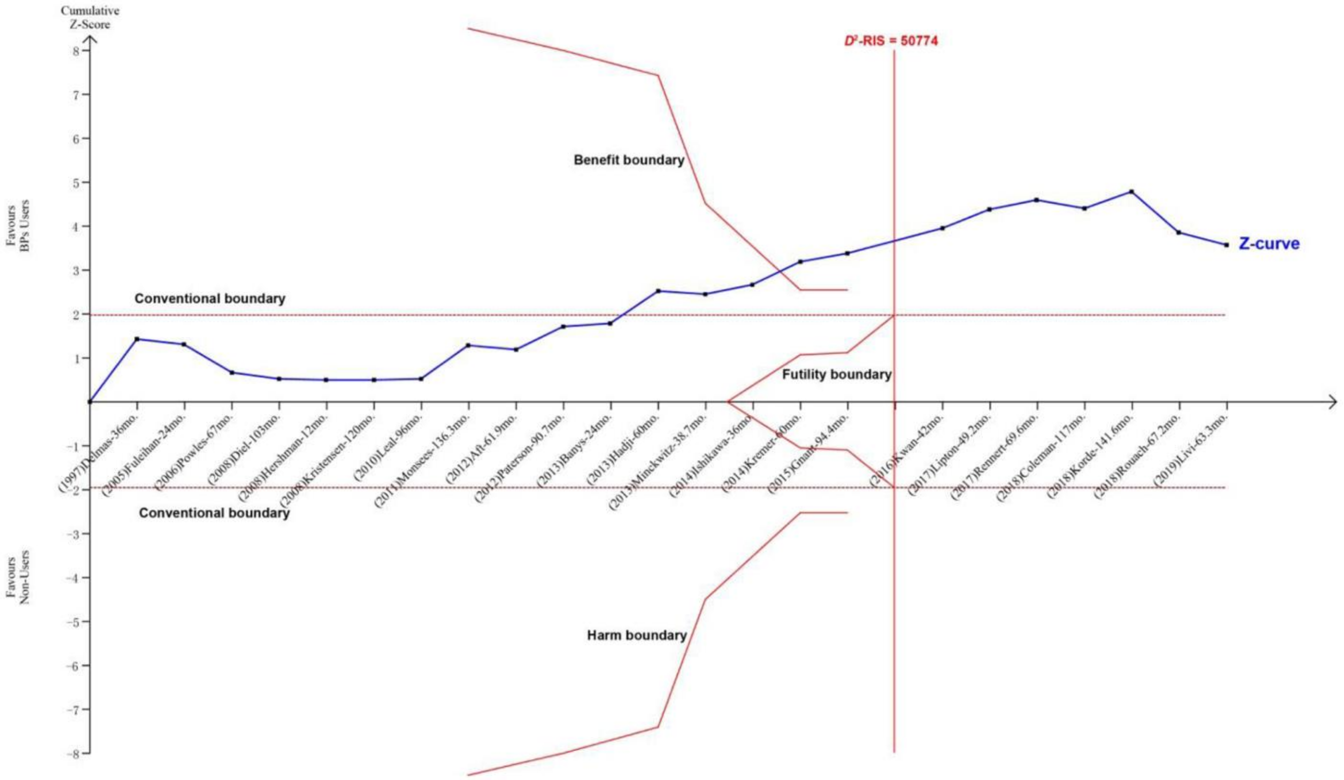

Supplementary Figure 3. Sensitivity analysis of bisphosphonates and breast cancer survival with trial sequential analysis with 90% statistical power.

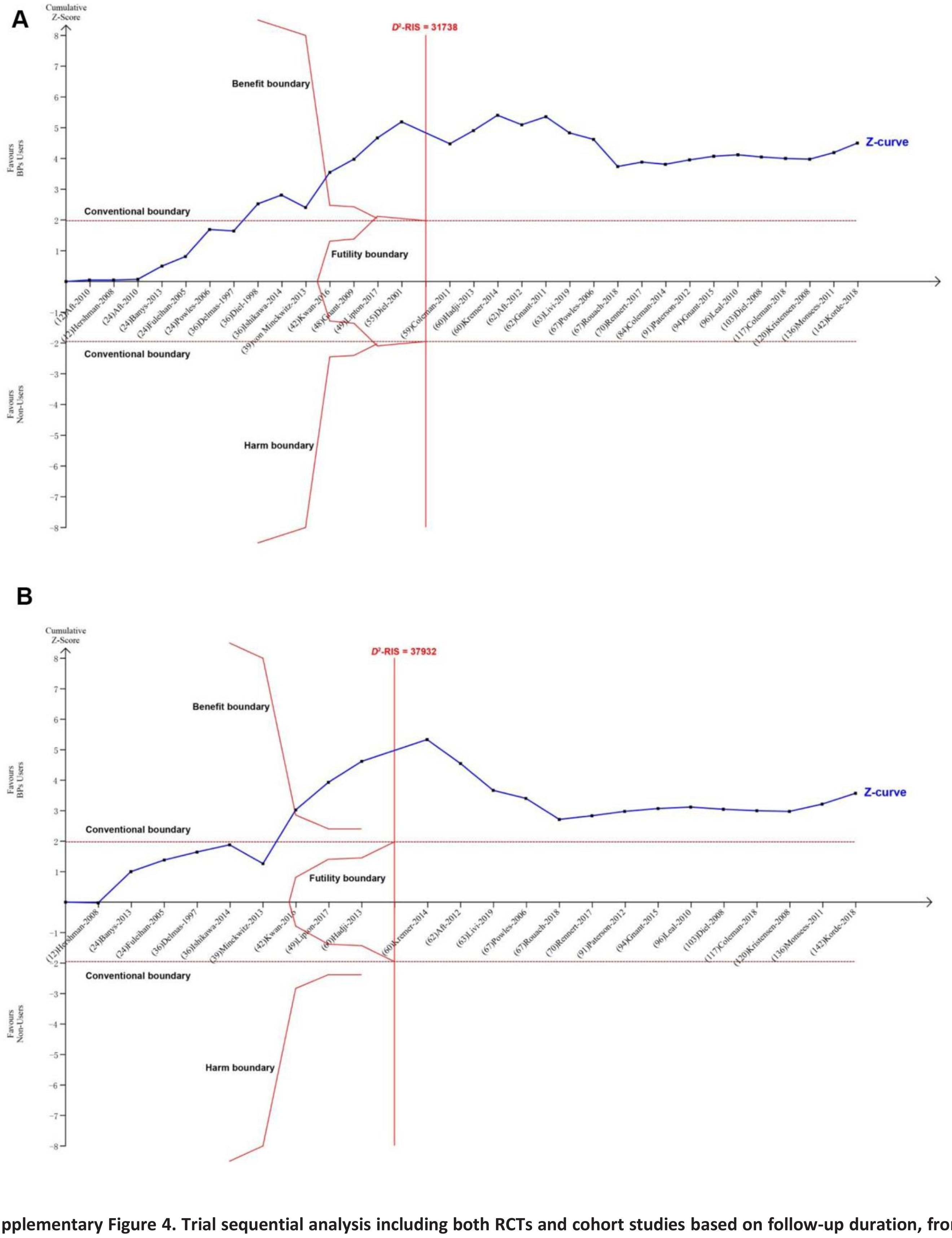

Supplementary Figure 4. Trial sequential analysis including both RCTs and cohort studies based on follow-up duration, from the shortest to longest among studies, irrespective of the treatment period. (**A**) Trial sequential analysis including all the multiple datasets at different follow-up time points from the same RCT; (**B**) Trial sequential analysis including only the dataset with the longest follow-up period from each individual RCTs.

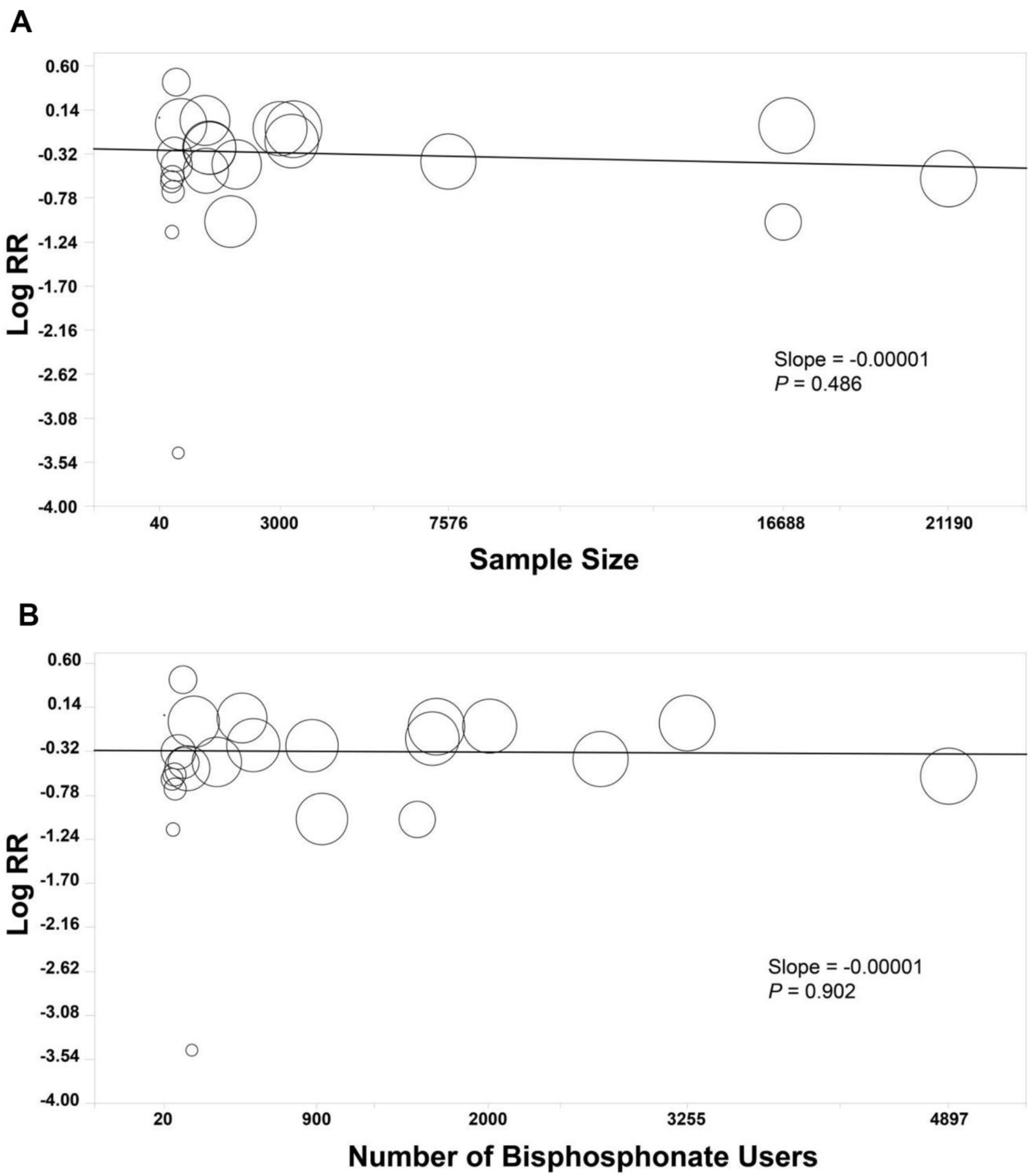
A
0.60
0.14
-0.32
-0.78
-1.24
-1.70
-2.16
-2.62
-3.08
-3.54
-4.00
Log RR
40
3000
7576
16688
21190
Sample Size
Slope = -0.00001
P = 0.486
B
0.60
0.14
-0.32
-0.78
-1.24
-1.70
-2.16
-2.62
-3.08
-3.54
-4.00
Log RR
20
900
2000
3255
4897
Number of Bisphosphonate Users
Slope = -0.00001
P = 0.902

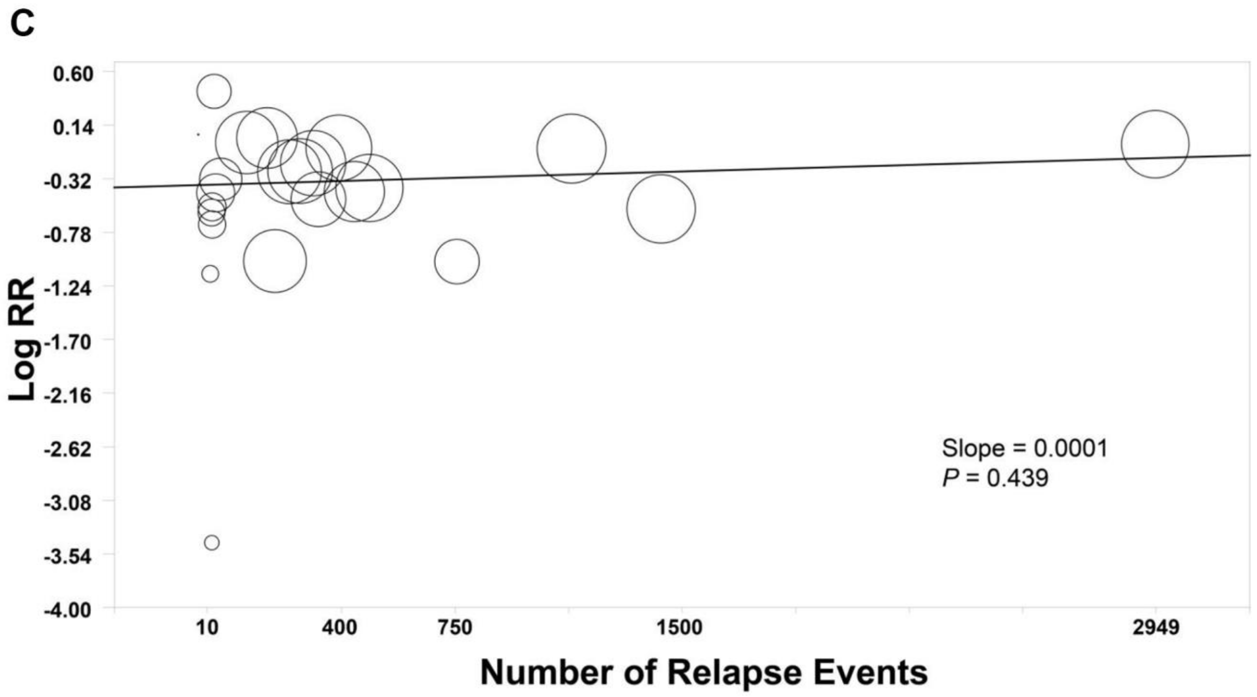

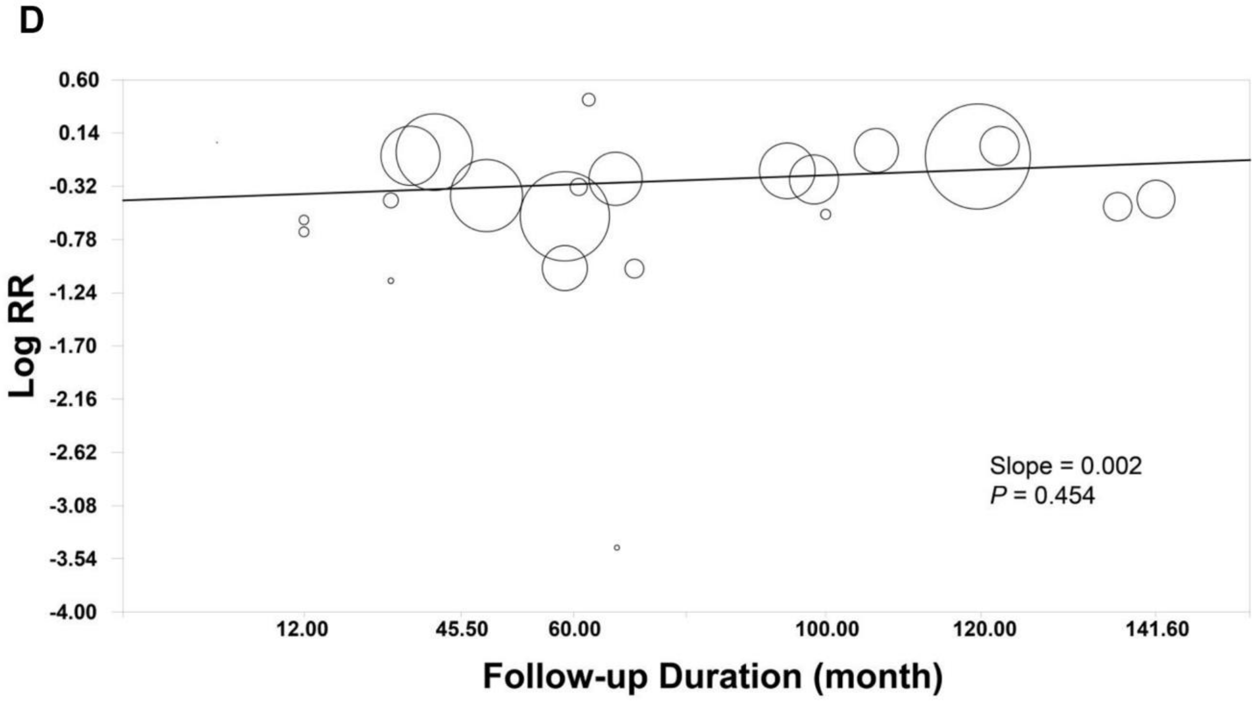

Supplementary Figure 5. Meta-regression analysis of bisphosphonates and breast cancer survival by (**A**) Sample size; (**B**) Number of bisphosphonate users; (**C**) Number of events; (**D**) Time period of follow-up duration in individual studies. Circles indicated individual studies, and the size of the circle was proportional to the relative weight that the study had in calculating the summary effect estimate.

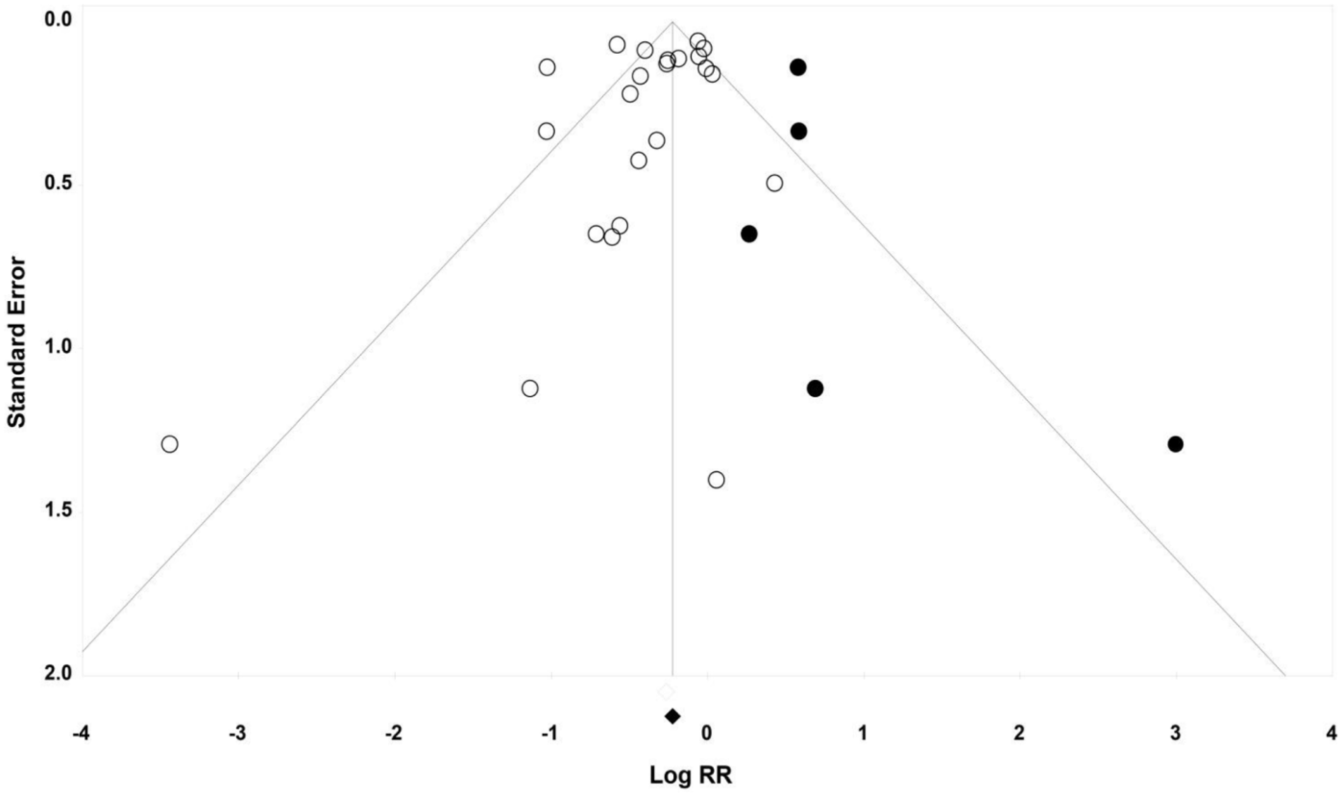

Supplementary Figure 6. Funnel plots of potential publication bias. White circles indicate individual studies; black circles, trimmed studies after adjustment for potential publication bias; white diamonds, the summarized effect estimates from the meta-analyses; black diamonds, the adjusted summary estimates. The diagonal lines represent the 95% confidence interval of the summarized effect estimate, which is indicated by the vertical line.
