# Supplementary material for: Bisphosphonates and breast cancer survival: a meta-analysis and trial sequential analysis of 81508 participants from 23 prospective epidemiological studies
Source: Aging (Albany NY). 2021 Aug 10;13(15):19835–66. doi: 10.18632/aging.203395 (PMC8386537; doi:10.18632/aging.203395)
Supplement: Supplementary Table 1 [file aging-13-203395-s003.docx]

**Supplementary Table 1. Characteristics of randomized controlled trials included in this study.**

| **Study,**  **Country** | **Study design,**  **Jadad score** | **Population characteristics, Median (range)/Mean age (SD), years** | **Study period (years),  Mean/median duration of follow-up (months)** | **Sample size BPs/Control group** | **Cancer therapy**  **(ACT, NACT, AET)** | **Compared Arms** | **Definition of BPs treatment (dose, frequency, and duration)** | **ER status  No. of cases (BPs/Control)** | **Menopausal status  No. of cases (BPs/Control)** | **Outcomes**  **/End-points** |
| --- | --- | --- | --- | --- | --- | --- | --- | --- | --- | --- |
| **Zoledric acid** |  |  |  |  |  |  |  |  |  |  |
| Gnant 2009  Austria | a multicenter, randomized,  non-placebo controlled trial, (ABCSG-12,  NCT00295646) | Premenopausal women who have a stage I/II hormone-positive breast cancer, and have ≤10 positive lymph nodes, but no evidence of recurrence, 45 (25-58) | 1999/2006-2008 47.8 | 900/903 | All patients received AET(goserelin plus either tamoxifen or anastrozole).  NACT was allowed, but none of the patients received ACT. | Zoledronic acid vs.  non-placebo control | Intravenous zoledronic acid 4 mg every 6 months for 3 years (total 6 doses)* | Positive: 840/850 Negative: 37/31 Unknown: 23/22 | All Premenopausal†  Pre: 200/213 Post: 700/690 | DFS, RFS, OS, bone metastasis-free survival |
| Gnant 2011 |  |  | 1999/2006-2010 62 (0–114.4) |  |  |  |  |  |  |  |
| Gnant 2011 |  |  | 1999/2006-2011 84/76 |  |  |  |  |  |  |  |
| Gnant 2015 |  |  | 1999/2006-2012  94.4 (0-114) |  |  |  |  |  |  |  |
| Aft 2010  the United States | a single center randomized,  non-placebo controlled trial, (NCT00242203) | Women with clinical stage II/III (≥T2 and/or ≥N1) newly histologically diagnosed breast cancer and no evidence of distant metastases, BPs: 50 (30–68); Control: 49.1 (32–69) | 2003/2006-2007 24/12 | 60/59 | All patients received NACT (epirubicin plus docetaxel and oral dexamethasone) and ACT (epirubicin plus docetaxel). | Zoledronic acid vs.  non-placebo control | Intravenous zoledronic acid 4 mg every 3 weeks for 1 years (total 17 doses) | Positive: 32/35 Negative: 28/24 | Pre: 31/33 Post: 29/26 | DTCs, DFS, OS |
| Aft 2012 |  |  | 2003/2006-2010 61.9 |  |  |  |  |  |  |  |
| Leal 2010  the United States | a randomized, non-placebo controlled trial, | Postmenopausal women with histologically confirmed stage II/III primary breast cancer with positive nodes, and no evidence of recurrence BPs: 54.5(41-83); Control: 50.5(37-65) | 2000/2007-2009 96 | 36/32 | Most of patients received ACT (94.1%, 64/68) or AET (88.2%, 60/68). | Zoledronic acid vs. non-placebo control | Intravenous zoledronic acid 4 mg every 12 weeks for 1 years (total 4 doses) | ER/PR positive: 29/29 ER and PR negative: 7/3 | All postmenopausal | BMD, DFS, OS |
| Coleman 2011  the United Kingdom | a multicenter, randomized,  non-placebo controlled trial, (AZURE/BIG-1-04, NCT00072020 /ISRCTN79831382) | Women with histologically confirmed primary breast cancer (T3-4 or N1), and no evidence of distant metastases, BPs: 51.6(9.9); Control: 51.3(10.0) | 2003/2006-2010 BPs: 59.3(53.5-60.9) Control: 58.6(52.7-60.9) | 1681/1678 | All patients received ACT (anthracyclines or taxanes) and/or AET. | Zoledronic acid vs.  non-placebo control | Zoledronic acid was administered intravenously in a dosage of 4 mg with 6 doses in the first 6 months, 8 doses in the following 24 months and 5 doses in the final 30 months (total 19 doses during 5 years) | Positive: 1319/1316 Negative: 349/355  Unknown: 13/7 | Pre: 751/752 ^‡^ Post: 766/766 Unknown: 164/160 | DFS, OS |
| Coleman 2014 |  |  | 2003/2006-2013  BPs: 84.0(69.7-93.2) Control: 84.0(63.3-92.2) |  |  |  |  |  |  |  |
| Coleman 2018 |  |  | 2003/2006-2016  117(70.4-120.4) |  |  |  |  |  |  |  |
| Banys 2013  Germany | a multicenter, randomized,  non-placebo controlled trial, (NCT00172068) | Women with histologically confirmed primary breast cancer (T1-4, N1-2, M0) and DTCs-positive bone marrow  BPs: 54 (36–71); Control: 54 (37–72) | 2002/2004-2011  88 (8–108) | 40/46 | All patients received ACT and/or AET. | Zoledronic acid vs.  non-placebo control | Intravenous zoledronic acid every 4 weeks for 24 months (total 96 doses during 2 years). | Positive: 30/30 Negative: 5/4 Unknown: 5/12 | Pre: 14/17 Post: 26/29 | DTCs, bone metastasis-free survival, DFS |
| Hershman 2008  the United States | a multicenter, double-blind,  randomized, placebo-controlled trial  (NCT00049452) | Premenopausal women were newly diagnosed nonmetastatic breast cancer.  BPs: 43(6); Control: 42(6) | 2001/2002-2004  24 | 50/53 | All patients received ACT. | zoledronic acid vs. placebo | Zoledronic acid 4 mg intravenously over 15 minutes every 3 months for 12 months (total 4 doses during 1 year) | ER/PR Positive: 37/37  ER and PR negative: 13/16 | All Premenopausal | BMD; Markers of Bone Turnover; any recurrence |
| Von Minckwitz 2016  Germany and Austria | a multicenter, open label, randomized, non-placebo clinical trial  The NaTaN study (NCT00512993) | Female BCa patients previously treated with NACT for at least four cycles, of which at least two cycles had to contain a taxane and an anthracycline and with completely resected unilateral or bilateral primary carcinoma of the breast with histologically detectable tumour residuals (T-4) and/or histology confirmed involvement of axillary nodes (N1-3), and no evidence of metastases (M0)  ≤55: 460 (66.4%); >55: 233 (33.6%) | 2005/2009-2014  54.7 | 343/350 | All patients received NACT. | zoledronic acid vs. observation | Zoledronic acid was given as a 15 min intravenous infusion at a dose of 4 mg every 4 weeks during the first 6 months, every 3 months for the next 2 years, and every 6 months for the last 2.5 years for a total of 19 infusions. The starting dose was reduced for patients showing a creatinine clearance below 60 ml/min. | ER/PR Positive: 269/279  ER and PR Negative: 73/70 | Pre: 99/86 Post: 237/250 | DFS, OS |
| Ishikawa 2014  Japan | a multicenter,open-lable, randomized, non-placebo controlled trial,  the JONIE1 study (UMIN000003261) | Women with histologically proven  invasive breast cancer of clinical stage IIA to IIIB (T≥3.0 cm and node negative, or T≥2.0 cm and cytologically or pathologically defined as node positive)  BPs: 49.5(34-71); Control: 49.0(28-70) | 2010/2012-2014  36 | 93/95 | All patients received NACT. | zoledronic acid vs. observation | Zoledronic acid (4 mg) was administered by intravenous infusion four times every 3 weeks and three times every 4 weeks during 1 year. | Positive: 71/75 Negative: 17/17 | Pre: 50/53 Post: 38/39 | pathologic complete response, DFS |
|  |  |  |  |  |  |  |  |  |  |  |
|  |  |  |  |  |  |  |  |  |  |  |
|  |  |  |  |  |  |  |  |  |  |  |
| **Supplementary Table 1. (Continued)** | |  |  |  |  |  |  |  |  |  |
| **Study,**  **Country** | **Study design,**  **Risk of bias (Low/High)** | **Population characteristics, Median (range)/Mean age (SD), years** | **Study period (years),  Mean/median duration of follow-up (months)** | **Sample size BPs/Control group** | **Cancer therapy**  **(ACT, NACT, AET)** | **Compared Arms** | **Definition of BPs treatment (dose, frequency, and duration)** | **ER status  No. of cases (BPs/Control)** | **Menopausal status  No. of cases (BPs/Control)** | **Outcomes**  **/End-points** |
| **Clodronate** |  |  |  |  |  |  |  |  |  |  |
| Diel 1998  Germany | a single center, randomized,  non-placebo controlled trial, | Women with histologically confirmed primary breast cancer (T1-4 and N0-2) , with positive immunocytochemical detection of at least one tumor cell in a bone marrow aspirate, but without distant metastasis, 51 (24-78) | 1990/1995-1997 36 | 157/145 | Most (81.5%, 246/302) of patients received ACT (cyclophosphamide, methotrexate, fluorouracil or epirubicin) and/or tamoxifen or goserelin. | Clodronate vs.  non-placebo control | Oral clodronate 1600 mg daily for 2 years | Positive: 104/84 Negative: 35/34 | Pre: 56/57 Post: 101/88 | the incidence and number of distant metastases (bone or visceral), OS |
| Diel 2001 |  |  | 1990/1995-1999 55 |  |  |  |  |  |  |  |
| Diel 2008 |  |  | 1990/1995-2004 103±12 |  |  |  |  |  |  |  |
| Saarto 2001  Finland | a single center, randomized, non-placebo controlled trial, | Women with histologically proven primary breast cancer with positive nodes, and no evidence of metastases (T1 to T3, N1/2, M0) 52 | 1990/1993-1999 60 | 139/143 | Premenopausal patients (53.9%, 152/282) received ACT (cyclophosphamide, methotrexate and fluorouracil) and postmenopausal patients (46.1%, 130/282) received only tamoxifen or torenifene. | Clodronate vs.  non-placebo control | Oral clodronate 1600 mg daily for 3 years | Positive: 70/86 Negative: 62/44 Unknown: 7/13 | Pre: 67/81 Post: 72/62 | metastases  -free survival, OS |
| Saarto 2004 |  |  | 1990/1993-2003 120 |  |  |  |  |  |  |  |
| Powles 2002  the United Kingdom, Canada, Norway, and Finland | a multicenter, double-blind, randomized, placebo-controlled trial (ISRCTN83688026) | Women with histologically or cytologically confirmed primary breast cancer, and no evidence of metastatic disease BPs: 52.8(10.6); Control: 52.7(10.5) | 1989/1995-2000 67 | 530/539 | Most (94.7%, 1012/1069) of patients received ACT (mitoxantrone, methotrexate, mitomycin, cyclophosphamide, fluorouracil, doxorubicin, epirubicin or cisplatin) and/or tamoxifen. | Clodronate vs.  placebo | Oral clodronate 1600 mg daily for 2 years | Positive: 245/240 Negative: 136/136 Unknown: 149/163 | Pre: 265/265 Post: 265/274 | time to first bone metastases, OS, the occurrence of nonskeletal relapses |
| Powles 2006 |  |  | 1989/1995-2000 67/24 |  |  |  |  |  |  |  |
| Paterson 2012  the United States | a multicenter, double-blind, randomized, placebo-controlled trial, (NSABP B-34, NCT00009945) | Women with histologically confirmed primary breast cancer and no evidence  of metastases, ≤49 yrs: 1183(35.6%);  ≥50 yrs: 2140(64.4%) | 2001/2004-2011 90.7 (IQR: 82.7–100.0) | 1655/1656 | Most (96.8%, 3164/3268) of patients received ACT (doxorubicin, cyclophosphamide, fluorouracil methotrexate or taxanes) and/or AET (tamoxifen, raloxofene, anastrozole, exemestane or letrozole). | Clodronate vs.  placebo | Oral clodronate 1600 mg daily for 3 years | 75% were ER-positive ER/PR positive: 1294/1293 ER and PR negative: 368/368 | Pre: 594/589 Post: 1068/1072 | DFS, OS, recurrence-free interval, bone metastasis-free interval |
| **Pamidronate** |  |  |  |  |  |  |  |  |  |  |
| Kristensen 2008  Denmark, Sweden and Iceland, | a multicenter, randomized, non-placebo controlled trial | Women with histologically confirmed resectable primary breast cancer and no evidence of distant metastases, 47.6 (≤39 -69 yrs) | 1990-1996  120 | 460/493 | All patients received ACT (cyclophosphamide, fluouracil, methotrexate or epirubicin). | Pamidronate vs.  non-placebo control | Oral pamidronate 150 mg twice daily for 4 years | Positive: 51/54 Negative: 135/136 Unknown: 274/303 | Pre: 308/326 Post: 152/166 Unknown: 0/1 | SREs, BMD, OS, occurrence of bone metastases |
| Fuleihan 2005  Lebanon | a single center, randomized, double-blind, placebo controlled trial, | Women with histologically proven, nonmetastatic breast cancer  40 (6) | 2000/2001-2004  24±9.6 | 21/19 | The individual ACT were chosen by their oncologist and not dictated by this study. | Pamidronate vs.  placebo control | Pamidronate 60 mg was administered by intravenous infusion every  3 months (0, 3, 6, and 9 months)  for 1 year. | NR. | All Premenopausal | BMD, occurrence of metastases, OS |
| **Ibandronate** |  |  |  |  |  |  |  |  |  |  |
| Von Minckwitz 2013  German | a multicenter, randomized, non-placebo controlled trial  (GAIN, NCT00196872) | Women with histologically confirmed node-positive primary breast cancer and no evidence of metastases (N1-2, M0,) 50(18-65) | 2004/2008-2011 38.7 (0-73.0) | 2015/1008 | All patients received ACT (epirubicine, cyclophosphamide, paclitaxel or capecitabine), with 50% were given a dose-dense regimen. | Ibandronate vs. non-placebo control | Oral ibandronate: 50 mg/day p.o. for 2 years | ER/PR positive: 1526/775 ER and PR negative: 470/222  Unknown: 0/1 | Pre: 961/470 Post: 1023/526 Unknown: 12/2 | DFS, OS, and others |
| Livi 2019  Italy | a single center, single-blind, randomized, placebo controlled trial,  (NCT02616744) | Women with histologically confirmed hormone receptorepositive early primary breast cancer, post-menopausal status,  60.2(44-75) | 2011/2014-2018 63.3 (2.7-87.3) | 89/82 | All patients received 5-year AET (exemestane, letrozole and anastrozole), vitamin D (4000 IU, weekly) and calcium (500 mg, daily) supplements. | Ibandronate vs. placebo control | Oral ibandronate: 150 mg/28 day p.o. for 2 years | NR. | All postmenopausal | The 2-year T-score of lumbar spine and total hip, DFS, OS |
| **Risedronate** |  |  |  |  |  |  |  |  |  |  |
| Delmas 1997  France | a single center, double-blind, randomized, placebo controlled trial, | Women with histologically confirmed breast cancer and artificially  induced menopause.  BPs: 45.7(4.0); Control: 46.6(4.6) | Study period: NR.  36 | 27/27 | All patients received ACT or radiotherapy. | Risedronate vs. placebo | Oral risedronate : daily drug (5mg) for 2 weeks, followed by 10 weeks without drug. Each cycle (12 weeks) was repeated 8 times over 96 weeks (for 2 years). | NR. | All postmenopausal | BMD, OS, relapse of breast cancer, |

**Abbreviations:** ABCSG-12, the Austrian Breast and Colorectal Cancer Study Group trial-12; ACT, adjuvant chemotherapy (postoperative chemotherapy); AET, adjuvant endocrine therapy; AZURE, the Adjuvant Zoledronic acid to redUce REcurrence trial; BIG, breast international group; BMD, bone mineral density; BPs, bisphosphonates; DFS, disease-free survival; DTCs, disseminated tumor cells; ER, estrogen receptor; GAIN, the German Adjuvant Intergroup Node Positive Study; HER2, Human Epidermal Growth Factor Receptor 2; IQR, inter quartile range; NACT, neoadjuvant chemotherapy (preoperative chemotherapy); NSABP B-34, the National Surgical Adjuvant Breast and Bowel Project protocol B-34; NR, not reported; OS, overall survival; Post, postmenopausal; PR, progesterone receptor; Pre, premenopausal; RFS, recurrence-free survival; SD, standard deviation; SREs, skeletal-related events.

*Zoledronic acid was given initially with a dosage of 8 mg every 4 weeks for 254 patients up to October 27, 2000, and then zoledronate was administered with 4 mg every 6 months.

†All the participants were premenopausal women and were given adjuvant ovarian suppression with goserelin; of whom, the subset of patients aged more than 40 years were more likely to achieve complete oestrogen deprivation as authors stated.
‡ Menopausal status was not identified prospectively in this trial; a cutoff at age 50 years to demarcate premenopause and postmenopause is a surrogate used frequently for menopausal status.
