# Supplementary material for: Bisphosphonates and breast cancer survival: a meta-analysis and trial sequential analysis of 81508 participants from 23 prospective epidemiological studies
Source: Aging (Albany NY). 2021 Aug 10;13(15):19835–66. doi: 10.18632/aging.203395 (PMC8386537; doi:10.18632/aging.203395)
Supplement: Supplementary Table 2 [file aging-13-203395-s004.docx]

**Supplementary Table 2. Characteristics of prospective epidemiological studies included in this study.**

| **Study,**  **Country** | **Study design,**  **Quality score, %** | **Population characteristics, Median (range)/Mean age (SD), years** | **Study period (years),  Mean/median duration of follow-up (months)** | **Sample size BPs/Control group** | **Cancer therapy** | **Compared Arms** | **Definition of BPs treatment (dose, frequency, and duration)** | **ER status  No. of cases (BPs/Control)** | **Menopausal status  No. of cases (BPs/Control)** | **Outcomes**  **/End-points** |
| --- | --- | --- | --- | --- | --- | --- | --- | --- | --- | --- |
|  |  |  |  |  |  |  |  |  |  |  |
| Hadji 2013  Germany | a historical cohort study  76 | Women with invasive breast cancer.  BPs users:59.5 (12.8);  Controls: 64.9 (13.4) | 1997-2009  60 | 962/684 | A majority of patients received with AET (98.4%) and ACT (61.3%). | Any BPs users vs. non-users  Zoledronate 314(34%)  Ibandronate 238(25%)  Alendronate 152(16%)  Clodronate 87(9%)  Others 146(16%) | Any BPs treatment within 1 year of breast cancer diagnosis and received ≥3 months of BPs treatment (zoledronic acid, clodronate, ibandronate, or alendronate; majority received zoledronic acid) | Positive: 778/509  Negative: 159/121 | NR. | DFS, OS |
| Korde 2018  the United States | a population-based cohort,  The Quilt cohort  82 | Women diagnosed with early-stage breast cancer.  64.2 (45-79) | 1993/1999-2015  141.6 (30-211.2) | 302/1511 | A majority of patients received with AET (66.8%; tamoxifen: 66.1%), radiation (66.7%), but not ACT (67.9%) | Any BPs users vs. non-users  Alendronate 272(90.0%)  Risendronate 40(13.0%)  Zoledronate 12(4.0%) | Any BPs used during the post-diagnosis period (women with no reported use and those with only one mention of use were classified as non-users)  Data sources of BPs prescription: from medical record reviews | Positive: 257/1269 Negative: 37/209 Unknown: 8/33 | Pre/Peri: 36/150  Post: 266/1261 | Recurrence of breast cancer (any, locoregional, distant), new breast primary, other cancer, breast cancer specific survival. |
| Kremer 2014  Canada | a historical cohort study  80 | Women with histologically confirmed primary breast cancer.  <65: 9112(42.3%); ≥65:12407 (57.7%)  (range: 40-≥80) | 1998/2005-2007  60 (0-120) | 4897/12286 | A majority of patients received with AET (62.3%; tamoxifen: 59.8%). | Any oral BPs users vs. non-users  (including alendronate, risedronate, and etidronate) | Oral BPs use during the pre-diagnosis and/or post-diagnosis period.  Data sources of BPs prescription: from the RAMQ database.  the Regie d’ssurance medicale de Québec | ER/PR Positive: 6927 Other/unknown: 14592 | Patients comprised predominantly of postmenopausal women. | Occurrence of bone metastases, OS, breast cancer specific survival. |
| Kwan 2016  the United States | a prospective cohort,  The KPNC and KPSC cohorts  87 | Women diagnosed with their first primary, early-stage breast cancer.  KPNC 59.6 (12.5)  BPs-users: 64.7 (11.0) Non-users: 58.8 (12.5)  KPSC 59.7 (12.3)  BPs-users: 64.5 (10.7) Non-users: 58.1 (12.3) | 1996/2007-2009  KPNC  BPs-users: 43.08 Non-users: 80.04  KPSC  BPs-users: 40.92 Non-users: 84.72 | KPNC: 1282/7575  KPSC: 1973/5951 | All patients treated with AET (tamoxifen). | Any BPs users vs. non-users  Alendronate: most commonly prescribed (>93 %) | Any BPs used during the post-diagnosis period (non-use: ≤90 days supply and use: >90 days supply)  Data sources of BPs prescription: from the Kaiser Permanente insurance records | KPNC:  ER/PR positive: 1016/6183 Other/unknown: 266/1392  KPSC:  ER/PR positive: 1207/3941 Other/unknown: 766/2010 | NR. | Occurrence of contralateral breast cancer, any recurrence with or without spread to regional areas or distant metastasis. |
| Lipton 2017  the United States | a prospective cohort,  the NCIC CTG MA.27 trial  69 | Women with histologically confirmed hormone receptor–positive primary invasive breast cancer.  64 | 2003/2008-2010  49.2 | 2711/4672 | All patients treated with AET.  (exemestane or anastrozole) | Any BPs users vs. non-users | Bisphosphonate use were reported as prior or present at the baseline. The length of the prior OPT therapy was unknown. In most cases but not all, the oral bisphosphonates used were noted. So we restricted bisphosphonate use to be at the baseline or at follow-up more than 30 days before disease recurrence. OPT had a variable duration and discretionary administration. | Positive:7525  Negative:51 | All postmenopausal | RFS, DFS |
| Monsees 2011  the United States | a nested case-control study from the SEER database  84 | Women with primary invasive, localized or regional SEER historic stage ER+ breast cancer  61.1 (40-79) | 1990/2005-2007  136.3 | 139/840 | A majority of patients received with AET (72.8%; tamoxifen: 64.8%), or radiation (65.5%), but not ACT (73.6%) | Any nitrogenous BPs  Alendronate:  106(88.0%) | Any nitrogenous BPs use was defined according to ever use, the recency of use, and the cumulative duration of use following the first breast cancer diagnosis.  Data sources of BPs prescription: the medical records of the oncology and/or primary care provider. | All ER positive | Case/control:  Pre: 45/86  Post: 199/417  Unknown: 107/159 | Occurrence of second primary contralateral breast cancer |
|  |  |  |  |  |  |  |  |  |  |  |
|  |  |  |  |  |  |  |  |  |  |  |
| **Supplementary Table 2. (Continued)** | |  |  |  |  |  |  |  |  |  |
| **Study,**  **Country** | **Study design,**  **Quality score, %** | **Population characteristics, Median (range)/Mean age (SD), years** | **Study period (years),  Mean/median duration of follow-up (months)** | **Sample size BPs/Control group** | **Cancer therapy** | **Compared Arms** | **Definition of BPs treatment (dose, frequency, and duration)** | **ER status  No. of cases (BPs/Control)** | **Menopausal status  No. of cases (BPs/Control)** | **Outcomes**  **/End-points** |
| Rennert 2017  Israel | A nested case–control study  from the population-based BCINIS cohort  76 | Women with breast cancer who reported being postmenopausal at time of diagnosis or were older than 55 years and who were CHS insurees.  Case: 69.7 (12.2)  Control: 67.2 (10.4) | 1998/2000-2014  69.6 | 1560/15128 | NR. | Second-generation BPs (alendronate and/or risedronate) | BPs users are defined as women whose alendronate and/or risedronate use was only after the diagnosis of breast cancer and before the index date.  Data sources of BPs prescription: from the CHS pharmacy records. | Case/control:  Positive: 579/13257  Negative/unknown: 194/2658 | All postmenopausal | OS, breast cancer specific survival |
| Rouach 2018  Israel | a historical cohort study  82 | Osteoporotic women diagnosed with early-stage breast cancer (breast cancer that has not spread beyond the breast or the axillary lymphnodes)  66.7 (10.4) | 2002/2012-2016  67.2 (45.6) | 145/81 | A majority of patients received with radiotherapy (69.6%), but not ACT (62.8%) | Any BPs users vs. non-users | Any BPs use of one-year duration, occurring at least one year before cancer diagnosis was considered as exposure.  Data sources of BPs prescription: from the medical file at Tel Aviv Medical Center. | Positive: 126/75 Negative: 18/6 Unknown: 1/0 | All postmenopausal | Occurrence of skeletal metastases |

**Abbreviations:** ACT, adjuvant chemotherapy (postoperative chemotherapy); AET, adjuvant endocrine therapy; BCINIS, the Breast Cancer in Northern Israel Study; BPs, bisphosphonates; CHS, Clalit Health Services; DFS, disease-free survival; ER, oestrogen receptor; KPNC, the Kaiser Permanente Northern California cohort; KPSC, the Kaiser Permanente Southern California cohort; NCIC CTG, the US National Cancer Institute and Canadian Cancer Trials Group; NR, not reported; OS, overall survival; Post, postmenopausal; PR, progesterone receptor; Pre, premenopausal; RFS, recurrence-free survival; SD, standard deviation; SEER, the Surveillance, Epidemiology, and End Results.
