# Supplementary material for: Bisphosphonates and breast cancer survival: a meta-analysis and trial sequential analysis of 81508 participants from 23 prospective epidemiological studies
Source: Aging (Albany NY). 2021 Aug 10;13(15):19835–66. doi: 10.18632/aging.203395 (PMC8386537; doi:10.18632/aging.203395)
Supplement: Supplementary Table 3 [file aging-13-203395-s005.docx]

**Supplementary Table 3. Methodological quality scores of prospective epidemiological studies (including cohort and nested case-control studies).**

| **Study name** | **Score of methodological quality** | | | | | | | | | | | | | | | | | | | |
| --- | --- | --- | --- | --- | --- | --- | --- | --- | --- | --- | --- | --- | --- | --- | --- | --- | --- | --- | --- | --- |
|  | **1 (0/4/8)** | **2 (0/4/7)** | **3 (0/4/7)** | **4 (0/10)** | **5 (0/8)** | **6 (0/4)** | **7 (0/4/6)** | **8 (0/2)** | **9 (0/6)** | **10 (0/4/6)** | **11 (0/4)** | **12 (0/4)** | **13 (0/8)** | **14 (0/6)** | **15 (0/4/9)** | **Selection bias  40**  **(1-5 items)** | **Misclassification bias 40**  **(6-13 items)** | **Confounding bias 15**  **(14-15 items)** | **Total** | **Reporting score (%)*** |
| **Cohort studies** |  |  |  |  |  |  |  |  |  |  |  |  |  |  |  |  |  |  |  |  |
| Hadji 2013  Germany | **4** | **7** | **7** | **10** | **8** | **4** | **6** | **2** | **6** | **6** | **0** | **0** | **8** | **0** | **4** | **36** | **32** | **4** | **72** | **76** |
| Korde 2018  the United States | **4** | **7** | **7** | **10** | **8** | **0** | **6** | **2** | **6** | **6** | **4** | **0** | **8** | **6** | **4** | **36** | **32** | **10** | **78** | **82** |
| Kremer 2014  Canada | **0** | **7** | **7** | **10** | **8** | **4** | **6** | **2** | **0** | **6** | **4** | **4** | **8** | **6** | **4** | **32** | **34** | **10** | **76** | **80** |
| Kwan 2016  the United States | **0** | **7** | **7** | **10** | **8** | **4** | **6** | **2** | **6** | **6** | **4** | **0** | **8** | **6** | **9** | **32** | **36** | **15** | **83** | **87** |
| Lipton 2017  the United States | **0** | **7** | **7** | **10** | **8** | **4** | **4** | **2** | **0** | **6** | **0** | **0** | **8** | **6** | **4** | **32** | **24** | **10** | **66** | **69** |
| Rouach 2018  Israel | **0** | **7** | **7** | **10** | **8** | **4** | **6** | **2** | **6** | **6** | **0** | **4** | **8** | **6** | **4** | **32** | **36** | **10** | **78** | **82** |
| **Nested case-control studies** |  |  |  |  |  |  |  |  |  |  |  |  |  |  |  |  |  |  |  |  |
| Monsees 2011  the United States | **4** | **4** | **7** | **10** | **8** | **0** | **6** | **2** | **6** | **6** | **4** | **0** | **8** | **6** | **9** | **33** | **32** | **15** | **80** | **84** |
| Rennert 2017  Israel | **0** | **4** | **0** | **10** | **8** | **4** | **6** | **2** | **6** | **6** | **4** | **4** | **8** | **6** | **4** | **22** | **40** | **10** | **72** | **76** |

*The quality score of each study was presented as a percentage of the maximum score, and studies with a score more than 60% were categorized as high-quality studies.

*This quality scoring system is modified from that developed by Voskuil and colleagues (Voskuil DW et al. Physical activity and endometrial cancer risk, a systematic review of current evidence. *Cancer Epidemiol Biomarkers Prev.* 2007;16:639-648) and used before as previously described (Liu YP et al. Bisphosphonates and primary breast cancer risk: an updated systematic review and meta-analysis involving 963,995 women. *Clin Epidemiol*. 2019;11:593-603).
