# Supplementary material for: Bisphosphonates and breast cancer survival: a meta-analysis and trial sequential analysis of 81508 participants from 23 prospective epidemiological studies
Source: Aging (Albany NY). 2021 Aug 10;13(15):19835–66. doi: 10.18632/aging.203395 (PMC8386537; doi:10.18632/aging.203395)
Supplement: Supplementary Table 4 [file aging-13-203395-s006.docx]

**Supplementary Table 4. GRADE quality of evidence.**

| **Certainty assessment** | | | | | | | **№ of patients** | | **Effect** | | **Quality of evidence** | **Importance** |
| --- | --- | --- | --- | --- | --- | --- | --- | --- | --- | --- | --- | --- |
| **№ of studies** | **Study design** | **Risk of bias** | **Inconsistency** | **Indirectness** | **Imprecision** | **Other considerations** | **Any BPs** | **Non-users** | **Relative (95% CI)** | **Absolute (95% CI)** |  |  |
| **Overall** | | | | | | | | | | | | |
| 23 | RCTs and Cohorts | not serious | not serious | not serious | not serious | none | 2302/22521 (10.2%) | 7189/58989 (12.2%) | **RR 0.725** (0.627 to 0.839) | **34 fewer per 1,000** (from 45 fewer to 20 fewer) | ⨁⨁⨁◯  MODERATE | CRITICAL |
| **Subgroup by study design** | | | | | | | | | | | | |
| **RCTs** | | | | | | | | | | | | |
| 15 | RCTs | not serious | not serious | not serious | not serious | none | 1440/7790 (18.5%) | 1382/6821 (20.3%) | **RR 0.892** (0.829 to 0.961) | **22 fewer per 1,000** (from 35 fewer to 8 fewer) | ⨁⨁⨁⨁ HIGH | CRITICAL |
| **Cohort Studies** | | | | | | | | | | | | |
| 8 | Cohorts | not serious | not serious | not serious | not serious | none | 862/14731  (5.9%) | 5808/52168 (11.1%) | **RR 0.570** (0.436 to 0.745) | **48 fewer per 1,000** (from 63 fewer to 28 fewer) | ⨁⨁⨁◯  MODERATE | CRITICAL |
| **Subgroup by Recurrence Sites** | | | | | | | | | | | | |
| **Bone metastases** | | | | | | | | | | | | |
| 12 | RCTs and Cohorts | not serious | not serious | not serious | not serious | none | 770/15968  (4.8%) | 1900/27002 (7.0%) | **HR 0.713** (0.602 to 0.843) | **20 fewer per 1,000** (from 27 fewer to 11 fewer) | ⨁⨁⨁◯  MODERATE | CRITICAL |
| **Nonskeletal metastases** | | | | | | | | | | | | |
| 9 | RCTs and Cohorts | not serious | not serious | not serious | not serious | none | 886/9739  (9.1%) | 897/10895  (8.2%) | **HR 0.883** (0.768 to 1.014) | **9 fewer per 1,000** (from 18 fewer to 1 more) | ⨁⨁⨁◯  MODERATE | IMPORTANT |
| **Loco-regional recurrence** | | | | | | | | | | | | |
| 10 | RCTs and Cohorts | not serious | not serious | not serious | not serious | publication bias suspected ^*^ | 508/12717  (4.0%) | 2692/25357 (10.6%) | **HR 0.887** (0.771 to 1.020) | **11 fewer per 1,000** (from 23 fewer to 2 more) | ⨁⨁⨁◯ MODERATE | IMPORTANT |
| **Contralateral breast recurrence** | | | | | | | | | | | | |
| 11 | RCTs and Cohorts | not serious | not serious | not serious | not serious | none | 225/12838 (1.8%) | 972/26171 (3.7%) | **HR 0.775** (0.651 to 0.922) | **8 fewer per 1,000** (from 13 fewer to 3 fewer) | ⨁⨁⨁◯  MODERATE | IMPORTANT |
| **Subgroup by Menopausal Status** | | | | | | | | | | | | |
| **Postmenopausal Women** | | | | | | | | | | | | |
| 12 | RCTs and Cohorts | not serious | not serious | not serious | not serious | none | 665/7302  (9.1%) | 1891/23462  (8.1%) | **RR 0.737** (0.640 to 0.850) | **21 fewer per 1,000** (from 29 fewer to 12 fewer) | ⨁⨁⨁◯  MODERATE | CRITICAL |
| **Non-postmenopausal Women** | | | | | | | | | | | | |
| 6 | RCTs and Cohorts | not serious | not serious | not serious | not serious | none | 523/2231  (23.4%) | 530/1949  (27.2%) | **RR 0.992** (0.864 to 1.139) | **2 fewer per 1,000** (from 37 fewer to 38 more) | ⨁⨁⨁◯  MODERATE | IMPORTANT |
| **Subgroup by Follow-up Duration** | | | | | | | | | | | | |
| **Treatment Duration** | | | | | | | | | | | | |
| 5 | RCTs | not serious | not serious | not serious | not serious | none | 446/2362  (18.9%) | 472/2376  (19.9%) | **RR 0.930** (0.817 to 1.058) | **13 fewer per 1,000** (from 33 fewer to 10 more) | ⨁⨁⨁⨁ HIGH | CRITICAL |
| **Post-treatment 1-2 years** | | | | | | | | | | | | |
| 8 | RCTs | not serious | not serious | not serious | not serious | none | 966/4929  (19.6%) | 882/3891  (22.7%) | **RR 0.780** (0.638 to 0.954) | **45 fewer per 1,000** (from 75 fewer to 9 fewer) | ⨁⨁⨁⨁ HIGH | CRITICAL |
| **Post-treatment 3-4 years** | | | | | | | | | | | | |
| 4 | RCTs | not serious | not serious | not serious | not serious | none | 240/1676  (14.3%) | 288/1669  (17.3%) | **RR 0.718** (0.585 to 0.881) | **45 fewer per 1,000** (from 68 fewer to 19 fewer) | ⨁⨁⨁⨁ HIGH | CRITICAL |
| **Post-treatment >4 years** | | | | | | | | | | | | |
| 7 | RCTs | not serious | not serious | not serious | not serious | none | 920/4948  (18.6%) | 1014/4965  (20.4%) | **HR 0.906** (0.832 to 0.987) | **17 fewer per 1,000** (from 31 fewer to 2 fewer) | ⨁⨁⨁⨁ HIGH | CRITICAL |
| **Subgroup by BP Type** | | | | | | | | | | | | |
| **Zoledronic acid** | | | | | | | | | | | | |
| 7 | RCTs | not serious | not serious | not serious | not serious | publication bias strongly suspected^†^ | 606/2855  (21.2%) | 666/2863  (23.3%) | **RR 0.892** (0.805 to 0.988) | **25 fewer per 1,000** (from 45 fewer to 3 fewer) | ⨁⨁⨁◯ MODERATE | IMPORTANT |
| **Clodronate** | | | | | | | | | | | | |
| 3 | RCTs | not serious | not serious | not serious | not serious | none | 339/2342  (14.5%) | 387/2340  (16.5%) | **RR 0.846** (0.736 to 0.971) | **25 fewer per 1,000** (from 44 fewer to 5 fewer) | ⨁⨁⨁⨁ HIGH | IMPORTANT |

**GRADE quality of evidence:** **High quality:** Further research is unlikely to have an important impact on confidence in estimate of effect and is likely to change the estimate; and we are very confident that the true effect lies close to that of the estimate of the effect. **Moderate quality:** Further research is possible to have a moderate impact on confidence in estimate of effect; we are moderately confident in the effect estimate; and the true effect is likely to be close to the estimate of the effect, but there is a possibility that it is substantially different. **Low quality:** Further research is likely to have an important impact on confidence in estimate of effect and is likely to change the estimate; our confidence in the effect estimate is limited; and the true effect may be substantially different from the estimate of the effect. **Very Low quality:** We have very little confidence in the effect estimate; and the true effect is likely to be substantially different from the estimate of effect.

Abbreviations: BCa, breast cancer; BPs, bisphosphonates; CI, conﬁdence interval; RCTs, randomized controlled trials; RR, risk ratio.

*Possibility for publication bias according to the Egger's linear regression test (P=0.006).

†Possibility for publication bias according to the Egger's linear regression test (P=0.042).
