# Supplementary material for: Bisphosphonates and breast cancer survival: a meta-analysis and trial sequential analysis of 81508 participants from 23 prospective epidemiological studies
Source: Aging (Albany NY). 2021 Aug 10;13(15):19835–66. doi: 10.18632/aging.203395 (PMC8386537; doi:10.18632/aging.203395)
Supplement: Supplementary Tables 5 and 6 [file aging-13-203395-s007.pdf]

SUPPLEMENTARY TABLES

Supplementary Table 5. Sensitivity analysis by comparison of the summarised results of the maximally and unadjusted effect estimates using confounding RR.

Subgroups	No. of datasets	Unadjusted effect estimates		Maximally adjusted effect estimates	Confounding RR	P-value for heterogeneity between results
		Pooled RR (95% CIs), *P*-value	I^2 (*P*-value)	Pooled RR (95% CIs)		
**Overall**	23	0.708 (0.607, 0.826), <0.001	75.73 (<0.001)	0.725 (0.627, 0.839)	1.023	0.836
**Study Design**						
RCTs	15	0.861 (0.782, 0.948), 0.002	0.00 (0.756)	0.892 (0.829, 0.961)	1.036	0.564
Cohorts	8	0.560 (0.398, 0.787), 0.001	88.13 (<0.001)	0.570 (0.436, 0.745)	1.018	0.939
**Follow-up Duration (year) (Only RCTs)**						
Treatment duration	5	0.723 (0.530, 0.986), 0.041	0.00 (0.866)	0.930 (0.817, 1.058)	1.286	0.143
Post-treatment 1-2	8	0.706 (0.535, 0.930), 0.013	44.32 (0.096)	0.780 (0.638, 0.954)	1.105	0.566
Post-treatment 3-4	4	0.694 (0.499, 0.966), 0.031	41.18 (0.183)	0.718 (0.585, 0.881)	1.035	0.865
Post-treatment >4	7	0.868 (0.764, 0.985), 0.028	0.00 (0.585)	0.906 (0.832, 0.987)	1.044	0.583
**Recurrence Sites**						
Bone metastases	12	0.748 (0.635, 0.881), 0.001	43.00 (0.063)	0.713 (0.602, 0.843)	0.953	0.688
Nonskeletal metastases	9	0.841 (0.730, 0.969), 0.016	17.04 (0.296)	0.883 (0.768, 1.014)	1.050	0.632
Loco-regional recurrence	10	0.835 (0.698, 0.999), 0.048	10.15 (0.351)	0.887 (0.771, 1.020)	1.062	0.603
Contralateral recurrence	11	0.725 (0.594, 0.885), 0.002	0.00 (0.569)	0.775 (0.651, 0.922)	1.069	0.628
**Menopausal Status**						
Postmenopausal	12	0.752 (0.636, 0.889), 0.001	37.02 (0.103)	0.737 (0.640, 0.850)	0.980	0.861
Non-postmenopausal	6	0.903 (0.685, 1.190), 0.468	16.01 (0.313)	0.992 (0.864, 1.139)	1.099	0.550
**Type of BPs (Only RCTs)**						
Zoledronic acid	7	0.738 (0.593, 0.920), 0.007	0.00 (0.978)	0.892 (0.805, 0.988)	1.209	0.126
Clodronate	3	0.864 (0.681, 1.097), 0.231	43.24 (0.184)	0.846 (0.736, 0.971)	0.979	0.878

*Confounding RR was defined as the ratio of the pooled results of the maximally adjusted and the unadjusted effect estimates. If the *P*-value for confounding RR was less than 0.05, the impact of adjustment for confounders on the pooled results was statistically significant; otherwise, the confounding effect of the adjusted factors was small or null. For protective factors, if the confounding RR <1, it indicates that the adjusted confounders have obscured an inverse association; if the confounding RR >1, it indicates that the adjusted confounders have exaggerated an inverse association, and therefore the summarized results of the maximally adjusted data are more conservative.

Abbreviations: BPs, bisphosphonates; CI, confidence interval; RCTs, randomized controlled trials; RR, risk ratio.

Supplementary Table 6. Sensitivity analysis by E-values for point estimates and upper confidence interval limits.

Subgroups	No. of datasets	Conventional meta-analysis			TSA		
		Pooled RR (95% CIs)	E-values for point estimate	E-values for Upper CI limit	TSA-corrected RR (95% CIs)	E-values for point estimate	E-values for Upper CI limit
**Overall**	23	**0.725 (0.627, 0.839)**	2.103	1.670	**0.700, (0.589, 0.832)**	2.211	1.695
**Study Design**							
RCTs	15	**0.892 (0.829, 0.961)**	1.490	1.246	**0.923, (0.856, 0.995)**	1.384	1.076
Cohorts	8	**0.570 (0.436, 0.745)**	2.905	2.020	**0.510, (0.318, 0.817)**	3.333	1.748
**Follow-up Duration (year) (Only RCTs)**							
Treatment duration	5	0.930 (0.817, 1.058)	1.360	1.000	0.905, (0.418, 1.960)	1.446	1.000
Post-treatment 1-2	8	**0.780 (0.638, 0.954)**	1.883	1.273	**0.774, (0.604, 0.992)**	1.906	1.098
Post-treatment 3-4	4	**0.718 (0.585, 0.881)**	2.132	1.527	0.798, (0.386, 1.653)	1.816	1.000
Post-treatment >4	7	**0.906 (0.832, 0.987)**	1.442	1.129	0.905, (0.798, 1.027)	1.446	1.000
**Recurrence Sites**							
Bone metastases	12	**0.713 (0.602, 0.843)**	2.154	1.656	**0.705, (0.527, 0.943)**	2.189	1.314
Nonskeletal metastases	9	0.883 (0.768, 1.014)	1.520	1.000	0.903, (0.684, 1.192)	1.452	1.000
Loco-regional recurrence	10	0.887 (0.771, 1.020)	1.506	1.000	0.639, (0.380, 1.075)	2.505	1.000
Contralateral recurrence	11	**0.775 (0.651, 0.922)**	1.902	1.388	**0.722, (0.606, 0.861)**	2.115	1.595
**Menopausal Status**							
Postmenopausal	12	**0.737 (0.640, 0.850)**	2.053	1.632	**0.737, (0.607, 0.895)**	2.053	1.479
Non-postmenopausal	6	0.992 (0.864, 1.139)	1.098	1.000	NA	NA	NA
**Type of BPs (Only RCTs)**							
Zoledronic acid	7	**0.892 (0.805, 0.988)**	1.490	1.123	0.920, (0.790, 1.072)	1.394	1.000
Clodronate	3	**0.846 (0.736, 0.971)**	1.646	1.205	0.891, (0.555, 1.431)	1.493	1.000

*E-value shows the minimum strength of association that a hypothetical residual confounding factor would need to have with both the use of bisphosphonates and the risk of BCa recurrence to fully explain the observed effect. Note that these calculations assumed an optimal frequency for the hypothetical confounder. For an infrequent confounder, the strength of the associations between the confounder and bisphosphonate use and between the confounder and BCa recurrence risk needs to be much stronger. In the present study, the impact of a hypothetical unmeasured confounder on the observed effect would be small or negligible when the E-value for the summarized point estimate is no less than 1.5.

Abbreviations: BPs, bisphosphonates; CI, confidence interval; RCTs, randomized controlled trials; RR, risk ratio; TSA, trial sequential analysis.
