# Supplementary material for: Bisphosphonates and breast cancer survival: a meta-analysis and trial sequential analysis of 81508 participants from 23 prospective epidemiological studies
Source: Aging (Albany NY). 2021 Aug 10;13(15):19835–66. doi: 10.18632/aging.203395 (PMC8386537; doi:10.18632/aging.203395)
Supplement: Supplementary Table 7 [file aging-13-203395-s008.docx]

**Supplementary Table 7. Sensitivity analysis by study design (RCTs vs. Cohorts).**

| **Subgroups** | **RCTs** | | | | |  | **Cohort study** | | | | | **Heterogeneity between study designs** |
| --- | --- | --- | --- | --- | --- | --- | --- | --- | --- | --- | --- | --- |
|  | **No. of datasets** | **Sample size of cumulative meta-analysis** | **Incidence in**  **control group (%)** | **Pooled RR (95% CIs), *P*-value** | ***I*^2^ (*P*-value)** |  | **No. of datasets** | **Sample size of cumulative meta-analysis** | **Incidence in**  **control group (%)** | **Pooled RR (95% CIs), *P*-value** | ***I*^2^ (*P*-value)** |  |
|  | | | | | | | | | | | |  |
| **Sub-total** | 15 | 14609 | 1382/6821 (20.25) | **0.892 (0.829, 0.961), 0.003** | 0.00 (0.722) |  | 8 | 66899 | 5808/52168 (11.13) | **0.570 (0.436, 0.745), <0.001** | 87.66(<0.001) | **0.002** |
| **Recurrence Sites** | | | | | | | | | | | |  |
| Bone metastases | 9 | 13978 | 561/6523 (8.60) | **0.801 (0.713, 0.899), <0.001** | 0.00 (0.914) |  | 3 | 28992 | 1339/20479 (6.54) | **0.504 (0.354, 0.718), <0.001** | 62.45 (0.070) | **0.015** |
| Nonskeletal metastases | 8 | 13058 | 740/6030 (12.27) | 0.953 (0.857, 1.059), 0.368 | 0.00 (0.480) |  | 1 | 7576 | 157/4856 (3.23) | **0.640 (0.474, 0.865), 0.004** | 0.00 (1.000) | **0.015** |
| Loco-regional recurrence | 7 | 11904 | 240/5455 (4.40) | 0.913 (0.765, 1.089), 0.311 | 0.00 (0.449) |  | 3 | 26170 | 2452/19902 (12.32) | 0.794 (0.556, 1.134), 0.205 | 54.48 (0.111) | 0.493 |
| Contralateral recurrence | 7 | 11860 | 96/5429 (1.77) | 0.870 (0.653, 1.159), 0.342 | 0.00 (0.810) |  | 4 | 27149 | 876/20742 (4.22) | **0.701 (0.521, 0.945), 0.020** | 43.96 (0.148) | 0.311 |
| **Menopausal Status** | | | | | | | | | | | |  |
| Postmenopausal | 8 | 4747 | 437/2127 (20.55) | **0.809 (0.717, 0.913), 0.001** | 0.00 (0.818) |  | 4 | 26017 | 1454/21335 (6.82) | **0.561 (0.378, 0.832), 0.004** | 66.80 (0.029) | 0.081 |
| Non-postmenopausal | 5 | 3894 | 440/1699 (25.87) | 1.015 (0.899, 1.147), 0.809 | 0.00 (0.909) |  | 1 | 286 | 90/250 (36.00) | **0.340 (0.121, 0.952), 0.040** | 0.00 (1.000) | 0.039 |

**Abbreviations:** CI, conﬁdence interval; RCTs, randomized controlled trials; RR, risk ratio.
