# Supplementary material for: Bisphosphonates and breast cancer survival: a meta-analysis and trial sequential analysis of 81508 participants from 23 prospective epidemiological studies
Source: Aging (Albany NY). 2021 Aug 10;13(15):19835–66. doi: 10.18632/aging.203395 (PMC8386537; doi:10.18632/aging.203395)
Supplement: Supplementary Table 8-12 [file aging-13-203395-s009.pdf]

Supplementary Table 8. Sensitivity analysis excluding the study that has the most relative weight in subgroups.

Subgroups	No. of datasets	Effect estimates after exclusion of the most relative-weight study				Before exclusion	P-value for difference between before and after exclusion	Excluded study (Relative weight within random effect models, %)
		Sample size of cumulative meta-analysis	Incidence in control group (%)	Pooled RR (95% CIs), *P*-value	I^2 (*P*-value)	Pooled RR (95% CIs)		
**Overall**	22	78151	6615/57311 (11.54)	0.708 (0.607, 0.826), <0.001	75.73 (<0.001)	0.725 (0.627, 0.839)	0.826	Coleman 2018-117mo.(7.70)
**Study Design**								
RCTs	14	11252	807/5143 (15.68)	0.861 (0.782, 0.948), 0.002	0.00 (0.756)	0.892 (0.829, 0.961)	0.568	Coleman 2018-117mo.(40.48)
Cohorts	7	45709	4644/36635 (12.68)	0.560 (0.398, 0.787), 0.001	88.13 (<0.001)	0.570 (0.436, 0.745)	0.936	Kremer 2014-60mo.(16.74)
**Follow-up Duration (year) (Only RCTs)**								
Treatment duration	4	1379	97/698 (13.83)	0.723 (0.530, 0.986), 0.041	0.00 (0.866)	0.930 (0.817, 1.058)	0.142	Coleman 2011-59.3mo.(82.62)
Post-treatment 1-2	7	5461	345/2213 (15.59)	0.706 (0.535, 0.930), 0.013	44.32 (0.096)	0.780 (0.638, 0.954)	0.568	Coleman 2014-84mo.(30.54)
Post-treatment 3-4	3	2276	143/1130 (12.65)	0.694 (0.499, 0.966), 0.031	41.18 (0.183)	0.718 (0.585, 0.881)	0.864	Powles 2006-67mo.(41.76)
Post-treatment >4	6	6554	439/3287 (13.36)	0.868 (0.764, 0.985), 0.028	0.00 (0.585)	0.906 (0.832, 0.987)	0.583	Coleman 2018-117mo.(54.22)
**Recurrence Sites**								
Bone metastases	11	21780	736/11469 (6.42)	0.748 (0.635, 0.881), 0.001	43.00 (0.063)	0.713 (0.602, 0.843)	0.689	Kremer 2014-60mo.(15.61)
Nonskeletal metastases	8	17275	537/9217 (5.83)	0.841 (0.730, 0.969), 0.016	17.04 (0.296)	0.883 (0.768, 1.014)	0.630	Coleman 2018-117mo.(22.66)
Loco-regional recurrence	9	21293	408/11831 (3.45)	0.835 (0.698, 0.999), 0.048	10.15 (0.351)	0.887 (0.771, 1.020)	0.603	Kwan 2016-42mo.(36.02)
Contralateral recurrence	10	22228	601/12645 (4.75)	0.725 (0.594, 0.885), 0.002	0.00 (0.569)	0.775 (0.651, 0.922)	0.621	Kwan 2016-42mo.(23.35)
**Menopausal Status**								
Postmenopausal	11	23188	1513/18597 (8.14)	0.752 (0.636, 0.889), 0.001	37.02 (0.103)	0.737 (0.640, 0.850)	0.857	Lipton 2017 -49.2mo.(19.88)
Non-postmenopausal	5	3018	530/1949 (27.17)	0.903 (0.685, 1.190), 0.468	16.01 (0.313)	0.992 (0.864, 1.139)	0.551	Coleman 2018 -117mo.(63.47)
**Type of BPs (Only RCTs)**								
Zoledronic acid	6	2359	129/1185 (10.84)	0.738 (0.593, 0.920), 0.007	0.00 (0.978)	0.892 (0.805, 0.988)	0.125	Coleman 2018 -117mo.(78.15)
Clodronate	2	1371	201/684 (29.53)	0.864 (0.681, 1.097), 0.231	43.24 (0.184)	0.846 (0.736, 0.971)	0.881	Paterson 2012 -90.7mo.(39.46)

Abbreviations: BPs, bisphosphonates; CI, confidence interval; RCTs, randomized controlled trials; RR, risk ratio.

Supplementary Table 9. Sensitivity analysis by including studies with high risk of bias (Saarto and Minckitz, RCT).

Subgroups	No. of datasets	Pooled RR (95% CIs), *P*-value	I^2 (*P*-value)
**Overall**	25	0.761 (0.657, 0.881), <0.001	79.99 (<0.001)
**Study Design**			
RCTs	17	0.920 (0.857, 0.987), 0.020	20.91 (0.210)
**Follow-up Duration (year) (Only RCTs)**			
Treatment duration	6	0.945 (0.838, 1.064), 0.350	0.00 (0.526)
Post-treatment 1-2	9	0.845 (0.668, 1.071), 0.163	66.75 (0.002)
Post-treatment >4	8	0.943 (0.821, 1.082), 0.403	46.48 (0.070)
**Recurrence Sites**			
Bone metastases	14	0.769 (0.646, 0.915), 0.003	68.96 (<0.001)
Nonskeletal metastases	10	0.935 (0.787, 1.110), 0.443	60.10 (0.007)
Loco-regional recurrence	12	0.898 (0.767, 1.051), 0.180	28.26 (0.168)
**Menopausal Status**			
Postmenopausal	13	0.743 (0.651, 0.849), <0.001	33.39 (0.115)
Non-postmenopausal	7	1.004 (0.894, 1.126), 0.951	0.00 (0.503)
**Type of BPs (Only RCTs)**			
Zoledronic acid	8	0.905 (0.821, 0.998), 0.045	0.00 (0.634)
Clodronate	4	0.966 (0.726, 1.284), 0.811	74.17 (0.009)

*Saarto and Minckitz studies are RCTs of high risk of bias and then are excluded from the primary main analyses, in order to minimize the impact on the overall results, especially the results of some subgroup analyses. However, sensitivity analysis showed that the inclusion of these studies did not materially impact the results.

Abbreviations: BPs, bisphosphonates; CI, confidence interval; RCTs, randomized controlled trials; RR, risk ratio.

Supplementary Table 10. Sensitivity analysis by excluding the zero-event study (Hershman, RCT).

Subgroups	No. of datasets	Pooled RR (95% CIs), *P*-value	I^2 (*P*-value)
**Overall**	22	0.724 (0.625, 0.838), <0.001	79.29 (<0.001)
**Study Design**			
RCTs	14	0.892 (0.829, 0.961), 0.003	0.00 (0.651)
**Follow-up Duration (year) (Only RCTs)**			
Treatment duration	4	0.885 (0.720, 1.088), 0.247	20.47 (0.287)
**Menopausal Status**			
Non-postmenopausal	5	0.973 (0.814, 1.161), 0.758	24.34 (0.259)
**Type of BPs (Only RCTs)**			
Zoledronic acid	6	0.892 (0.805, 0.988), 0.029	0.00 (0.493)

*Hershman' study is a randomized controlled trial, in which none event had been observed in both arms. The Hershman trial was only included in these analyses listed above.

Abbreviations: BPs, bisphosphonates; CI, confidence interval; RCTs, randomized controlled trials; RR, risk ratio.

Supplementary Table 11. Sensitivity analysis by excluding studies without clear definition of post-diagnosis BPs use (Lipton and Rouach, Cohort).

Subgroups	No. of datasets	Pooled RR (95% CIs), *P*-value	I^2 (*P*-value)
**Overall**	21	0.736 (0.631, 0.858), <0.001	78.38 (<0.001)
**Study Design**			
Cohorts	6	0.568 (0.408, 0.792), 0.001	90.21 (<0.001)
**Recurrence Sites**			
Bone metastases	12	0.750 (0.642, 0.877), <0.001	53.04 (0.024)
Nonskeletal metastases	8	0.953 (0.857, 1.059), 0.368	0.00 (0.480)
Loco-regional recurrence	9	0.937 (0.828, 1.060), 0.302	0.00 (0.528)
Contralateral recurrence	10	0.818 (0.681, 0.982), 0.031	0.00 (0.754)
**Menopausal Status**			
Postmenopausal	10	0.775 (0.686, 0.876), <0.001	8.03 (0.368)

*Lipton and Rouach studies are all cohort studies and are included in these analyses above, so this sensitivity analysis was conducted in such subgroup analyses listed above.
Abbreviations: CI, confidence interval; RR, risk ratio.

Supplementary Table 12. Publication bias analysis.

Subgroups	No. of datasets	*P*-value for		Pooled RR (95% CI)	
		Begg's rank correlation test	Egger's linear regression test	Adjusted with trim and fill	Unadjusted
**Overall**	23	0.091	0.259	0.796 (0.678, 0.933)	0.725 (0.627, 0.839)
Study Design					
RCTs	15	0.428	0.113	0.898 (0.834, 0.967)	0.892 (0.829, 0.961)
Cohorts	8	0.386	0.251	0.687 (0.520, 0.907)	0.570 (0.436, 0.745)
**Follow-up Duration (year) (Only RCTs)**					
Treatment duration	5	0.807	0.369	0.936 (0.823, 1.064)	0.930 (0.817, 1.058)
Post-treatment 1-2	8	0.548	0.195	0.786 (0.672, 0.920)	0.780 (0.638, 0.954)
Post-treatment 3-4	4	1.000	0.590	0.690 (0.544, 0.875)	0.718 (0.585, 0.881)
Post-treatment >4	7	0.711	0.258	0.906 (0.820, 1.001)	0.906 (0.832, 0.987)
**Recurrence Sites**					
Bone metastases	12	0.373	0.751	0.652 (0.549, 0.775)	0.713 (0.602, 0.843)
Nonskeletal metastases	9	0.917	0.352	0.931 (0.798, 1.087)	0.883 (0.768, 1.014)
Loco-regional recurrence	10	0.074	0.006	0.914 (0.801, 1.044)	0.887 (0.771, 1.020)
Contralateral recurrence	11	1.000	0.891	0.771 (0.648, 0.918)	0.775 (0.651, 0.922)
**Menopausal Status**					
Postmenopausal	12	0.193	0.270	0.746 (0.633, 0.878)	0.737 (0.640, 0.850)
Non-postmenopausal	6	0.260	0.134	1.013 (0.810, 1.266)	0.992 (0.864, 1.139)
**Type of BPs (Only RCTs)**					
Zoledronic acid	7	1.000	0.042	0.903 (0.816, 0.999)	0.892 (0.805, 0.988)
Clodronate	3	1.000	0.324	0.846 (0.736, 0.971)	0.846 (0.736, 0.971)

Abbreviations: BPs, bisphosphonates; CI, confidence interval; RCTs, randomized controlled trials; RR, risk ratio.
